# Tiglon enables accurate transcriptome assembly via integrating mappings of different aligners

**DOI:** 10.1016/j.isci.2022.104067

**Published:** 2022-03-12

**Authors:** Xiaoyu Zhao, Ting Yu

**Affiliations:** 1 Research Center for Mathematics and Interdisciplinary Sciences, Shandong University, Qingdao 266237, China; 2 School of Mathematics, Shandong University, Jinan, Shandong 250100, China

**Keywords:** Biological sciences, Bioinformatics, Systems biology, Experimental models in systems biology, Biological sciences research methodologies

## Abstract

Full-length transcript reconstruction has a pivotal role in RNA-seq data analysis. In this research, we present a new genome-guided transcriptome assembly algorithm, namely Tiglon, which integrates multiple alignments of different mapping tools and builds the labeled splice graphs, followed by a label-based dynamic path-searching strategy to reconstruct the transcripts. We evaluate Tiglon on a simulated dataset and 12 real datasets under the Hisat2 and Star mappings. The results indicate that the integrating techniques of Tiglon exhibit great superiority over the state-of-the-art assemblers, including StringTie2 and Scallop, depending on Hisat2 alignments, Star alignments, or the merged alignments of both. Especially, Tiglon is significantly powerful in recovering lowly expressed transcripts.

## Highlights

• Tiglon is designed for integrating multiple alignments to assemble transcripts
• Integrating alignments of different aligners is helpful for transcriptome assembly
• Tiglon proposes a new graph model called the labeled splice graph
• Our experiments demonstrate that Tiglon outperforms the leading assemblers

## Graphical abstract

**Figure:**
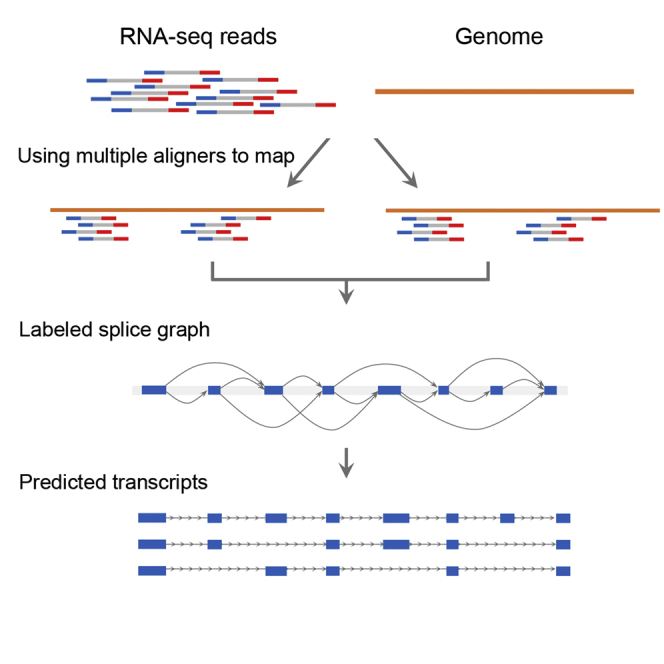

## Introduction

RNA-seq, as a powerful technology for transcriptome analysis, is extensively used worldwide. Especially during the past five years, this technology has transitioned from research to clinical use (Phillips et al., 2020), which sheds light on the study of complex diseases related to abnormal splicing events or differential expression levels such as cancers. Moreover, it provides the opportunity to view the complexity of eukaryotic transcriptomes, identify the expressed transcripts, and quantify their expression abundance precisely at a whole transcriptome level (Marguerat and Bähler, 2010; Ozsolak and Milos, 2010; Wang et al., 2009; Wilhelm and Landry, 2009). One of the most critical steps for RNA-seq data analysis is accurately assembling the tremendous amount of sequencing reads into full-length transcripts, which is quite a computationally challenging task.

The explosive growth of RNA-seq data has been driving the development of algorithms for transcriptome assembly. Quite a few algorithms have been developed for assembling RNA-seq reads into full-length transcripts. They are usually categorized into two strategies, *de novo* and genome-guided. *De novo* assemblers usually attempt to find overlaps between the RNA-seq reads and employ an extension technique to reconstruct the full-length transcripts. Such an approach is usually used where the reference genome is unavailable; therefore, this strategy mostly produces highly fragmented and error-prone transcripts. The state-of-the-art *de novo* assemblers include TransLiG (Liu et al., 2019), BinPacker (Liu et al., 2016a), Bridger (Chang et al., 2015), Trinity (MacManes and Eisen, 2013), ABySS (Simpson et al., 2009), SOAPdenovo-Trans (Xie et al., 2014), and IDBA-Tran (Peng et al., 2013). On the contrast, if a high-quality reference genome is available for model species, such as human, genome-guided assemblers such as StringTie2 (Kovaka et al., 2019), StringTie (Pertea et al., 2015), Scallop (Shao and Kingsford, 2017), TransComb (Liu et al., 2016b), TransBorrow (Yu et al., 2020b), Cufflinks (Trapnell et al., 2010), CLASS2 (Song et al., 2016), iPAC (Yu et al., 2020a), Traph (Tomescu et al., 2013), CEM (Li and Jiang, 2012), IsoLasso (Li et al., 2011), and Bayesembler (Maretty et al., 2014) can be employed. Such approaches generally first use the aligners such as Hisat2 (Kim et al., 2019), Hisat (Kim et al., 2015), Star (Dobin et al., 2013), Tophat2 (Kim et al., 2013), Tophat (Trapnell et al., 2009), SpliceMap (Au et al., 2010), MapSplice (Wang et al., 2010), or GSNAP (Wu and Nacu, 2010) to map the RNA-seq reads to the reference genome. Then, based on the alignments, a splice graph model is built for each gene locus, followed by employing different computational models to generate transcript-representing paths via traversing the graph. Genome-guided approaches usually show much higher accuracy than the *de novo* ones. Moreover, a number of tools are also developed to merge assemblies from multiple RNA-seq samples, such as the *merge* mode of StringTie2 (Kovaka et al., 2019) and TACO (Niknafs et al., 2017).

**Figure 1:**
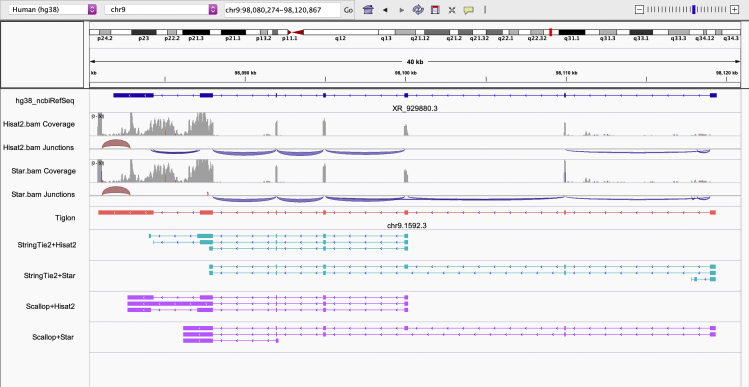
An IGV snapshot shows that the reference transcript named “XR_929,880.3” of human genome GRCh38 is covered by reads from RNA-seq sample SRA: SRR307911 The exons of this transcript are all captured by both Hisat2 and Star mappings, while its first junction is not captured by Star mapping, and its fifth junction is not captured by Hisat2 mapping. Depending on only one aligner, StringTie2 and Scallop cannot recover this transcript, while Tiglon recovers it by integrating both alignments.

To the best of our knowledge, none of the current assemblers is specifically designed for integrating alignments generated by different mapping tools, which is of great significance practically. As shown in the IGV snapshot (Figure 1), the annotated transcript “XR_929,880.3” in NCBI RefSeq from human reference genome GRCh38 is captured by the RNA-seq sample SRA: SRR307911 (NCBI SRA accession code). In the region, we can see that both the Hisat2 and Star (two of the best current aligners) alignments cover the whole exons of this transcript, but neither of them covers the entire junctions. Consequently, assemblers depending on only one aligner cannot recover this transcript, which is actually reconstructed by the proposed Tiglon algorithm. It suggests that integrating alignments of different aligners would be a helpful and meaningful strategy for transcriptome assembly.

In this research, we introduce Tiglon, an elaborately designed genome-guided transcriptome assembly approach that integrates mappings produced by different aligners. Taking advantage of different alignments, Tiglon builds a new graph model, namely labeled splice graph, in which each node corresponds to an exon, and each edge corresponds to a junction, and Tiglon further labels the edges (junctions) with 1 and 2, where label 2 indicates that they are captured by all the employed aligners and label 1 otherwise. In addition, label 2 further categorizes into 2+ and 2-, where 2 + means that the captured reads of different aligners are exactly the same and 2- otherwise. Next, based on the labeled splice graph, Tiglon employs a newly developed labeled-based dynamic path extension program to recover the expressed transcripts accurately.

Tested on a simulated dataset and 12 real datasets (8 from *Homo sapiens* samples and 4 from *Mus musculus* samples), the integrating techniques of Tiglon demonstrate significantly superior over the best current approaches, including StringTie2 and Scallop that depends on Hisat2 alignments, Star alignments, or the merged alignments of the two ones mentioned above, and StringTie2-Merge (the --*merge* mode of StringTie2). For instance, on the eight tested *H. sapiens* datasets, Tiglon averagely reconstructed 9.51% more correct transcripts than StringTie2-Merge, 11.25%–13.68% more than StringTie2 and Scallop depending on the merged alignments, and 19.82%–25.61% more than StringTie2 and Scallop depending on Hisat2 or Star alignments. More critically, Tiglon exhibits significant superiority over others in recovering lowly expressed transcripts. On the eight *H. sapiens* samples, Tiglon correctly recovered 22.57%–53.48% more lowly expressed transcripts than StirngTie2-Merge, 29.73%–89.05% more than Scallop and StringTie2 depending on the merged alignments, and 51.93%–161.18% more than StringTie2 and Scallop depending on only one aligner.

## Results

Tiglon is a transcriptome assembly approach that takes advantage of multiple alignments generated by different mapping tools. In this study, we first focused on the performance comparison between Tiglon and two of the best and representative assemblers StringTie2 and Scallop, depending on the Hisat2 alignments, Star alignments, and the merged alignments of both. Additionally, we evaluated the performance of Tiglon that was run with multiple aligners or with only one aligner, and we also compared Tiglon with two of our previous algorithms TransBorrow and iPAC. Based on the comparing results, the integrating strategy of Tiglon exhibited superior performance on both simulated and real datasets. The common comparison criteria used in this study were that a reference transcript is considered to be correctly identified if and only if its intron chain is exactly matched with an assembled transcript, and this matched assembled transcript is regarded as correctly assembled. And, we used the Cuffcompare tool in the Cufflinks package (Trapnell et al., 2010) to detect the correctly assembled transcripts. The accuracy of an assembler is measured by the number of correctly assembled transcripts and precision, the percentage of correctly assembled transcripts out of all the predicted ones. The versions of reference genome and transcriptome for the *H. sapiens* and *M. musculus* samples used in this research are GRCh38/hg38 and GRCm38/mm10, respectively, which were downloaded from the UCSC Genome Browser.

### Performance evaluation on simulated dataset

In this study, we used RNA-seq data simulator RSEM (Li and Dewey, 2011) to generate a simulated dataset that contained ∼52 million 100-bp length paired-end reads. The parameters of the simulation model were learned from the real human RNA-seq dataset with the NCBI SRA accession code of SRR7536920. Next, we mapped the simulated reads to the reference genome by using Hisat2 and Star. We subsequently used the *samtools merge* (Li et al., 2009) to generate the merged alignments based on the mapping results of the two aforementioned aligners. We ran Tiglon with alignments generated by both aligners as its input, while ran StringTie2 and Scallop with the alignments of each aligner and the merged alignments as their input, denoted as StringTie2+Hisat2, StringTie2+Star, StringTie2+MergedAlignments, Scallop + Hisat2, Scallop + Star, and Scallop + MergedAlignments. In addition, we ran StringTie2 in the mode *--merge* with the assemblies produced by StringTie2+Hisat2 and StringTie2+Star as its input to generate a unified set of transcripts, denoted as StringTie2-Merge.

**Figure 2:**
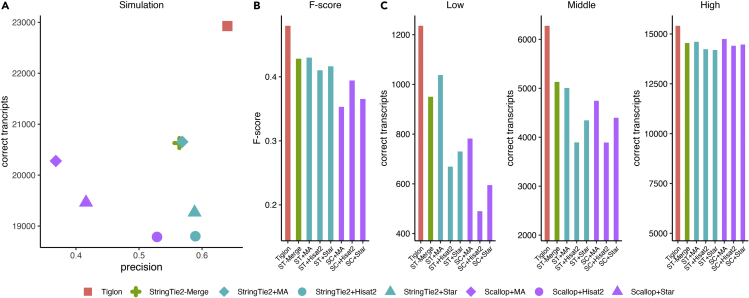
Performance evaluation on the simulated dataset (A) Precision and the number of correctly assembled transcripts of the assemblers on the simulated dataset. (B) F-score of the assemblers on the simulated dataset. (C) Comparisons of detected transcripts with low, middle, and high expression levels on the simulated dataset. The abbreviation ST is for StringTie2, SC for Scallop, and MA for MergedAlignments.

First, we evaluated the performance of each assembler by precision and the number of correctly assembled transcripts. Testing results on the simulated dataset showed that Tiglon performed the best, which correctly reconstructed much more transcripts, while kept the highest precision. Concretely, the correctly assembled transcripts of Tiglon reached 22,928, versus 20,630 for StringTie2-Merge, 20,654 for StringTie2+MergedAlignments, 20,277 for Scallop + MergedAlignments, while 18,800 for StringTie2+Hisat2, 19,267 for StringTie2+Star, 18,786 for Scallop + Hisat2, and 19,463 for Scallop + Star. Broadly, by making strategic use of the alignments of both aligners, Tiglon recovered 11.1% more correct transcripts than StringTie2-Merge, 11.01% more than StringTie2+MergedAlignments, 13.1% more than Scallop + MergedAlignments, while 17.8%–22% more than StringTie2 and Scallop depending on different aligners (Figure 2A and Table S2). Comparison results showed that Tiglon also achieved the highest precision of 64.01%, versus StingTie2-Merge of 56.40%, StringTie2+MergedAlignments of 56.87%, Scallop + MergedAlignments of 36.81%, StringTie2+Hisat2 of 58.94%, StringTie2+Star of 58.82%, Scallop + Hisat2 of 52.86%, and Scallop + Star of 41.60%. On these grounds, Tiglon showed superior performance among all the compared assemblers regardless of the number of correctly assembled transcripts or precision.

Besides, we further calculated the F-score, a harmonic mean of recall and precision (calculated as 2∗precision∗recall/(precision + recall)) to evaluate the overall performance of each assembler, where the recall means the fraction of correctly identified reference transcripts in the ground truth. On the simulated dataset, Tiglon obtained an F-score of 0.480, a significant increase over StringTie2-Merge of 0.428, StringTie2+MergedAlignments of 0.430, Scallop + MergedAlignments of 0.353, StringTie2+Hisat2 of 0.410, StringTie2+Star of 0.412, Scallop + Hisat2 of 0.394, and Scallop + Star of 0.365, which demonstrated that Tiglon had a more remarkable capability to balance the recall and precision (Figure 2B and Table S2).

Generally speaking, it is more difficult to reconstruct transcripts with relatively low expressions, while lowly expressed ones may play important roles in organisms. We then evaluated the ability of assemblers in recovering transcripts with different expression levels. As did by Shao et al. in their evaluation of Scallop (Shao and Kingsford, 2017), we first sorted the expressed transcripts according to their expression abundances. Then, all the expressed transcripts were equally divided into three parts, which corresponded to low, middle, and high expressed ones. Finally, we computed the number of correctly identified transcripts in each part for each assembler. The results revealed that Tiglon consistently outperformed all the others in recovering transcripts of different expression levels (Figure 2C and Table S15). Particularly for the lowly expressed ones, Tiglon correctly recovered 30.11% more than StringTie2-Merge, 19.08% more than StringTie2+MergedAlignments, 58.06% more than Scallop + MergedAlignments, and 40%–108% more than StringTie2 and Scallop depending on different aligners.

Based on the above comparison, it is concluded that by integrating alignments produced by Hisat2 and Star, Tiglon achieved the best performance among the tested assemblers. Especially, Tiglon reconstructed significantly more expressed transcripts than the others.

### Performance evaluation on the real datasets

Because the expressed transcripts and their expression abundances are precisely known for the simulated dataset, tests on the simulated data are persuasive. However, simulation cannot capture the entire features of real biological datasets, so evaluation on real datasets is of great significance to further verify the assembling performance in real applications. Different from the simulated dataset, the ground truth of real datasets is difficult to know. Nonetheless, it is generally safe to assume that an assembler is more accurate if it recovers more known annotated transcripts (Kovaka et al., 2019). In this study, all the transcripts (NCBI RefSeq in GTF format) of the species *H. sapiens* and *M. musculus* downloaded from the UCSC Genome Browser were set as the ground truth. And, eight *H. sapiens* RNA-seq samples H1–H8 and four *M. musculus* RNA-seq samples M1–M4 were collected to evaluate the performance of the assemblers. All these datasets were downloaded from NCBI Sequence Read Archive (SRA); the accession codes for the *H. sapiens* samples were SRA: SRR307911, SRA: SRR387662, SRA: SRR10517380, SRA: ERR2403203, SRA: SRR307903, SRA: SRR315323, SRA: SRR315334, and SRA: SRR7536920, and for the *M. musculus* samples were SRA: DRR205674, SRA: DRR205677, SRA: ERR3320855, and SRA: ERR3320871. The detailed description of these datasets can be found in Table S1. We then evaluated the assemblers on the 12 real datasets in terms of the same criteria as we did on the simulated dataset. The results exhibited that Tiglon consistently achieved the best performance on all the 12 real datasets.

#### Performance evaluation on the *H. sapiens* samples

**Figure 3:**
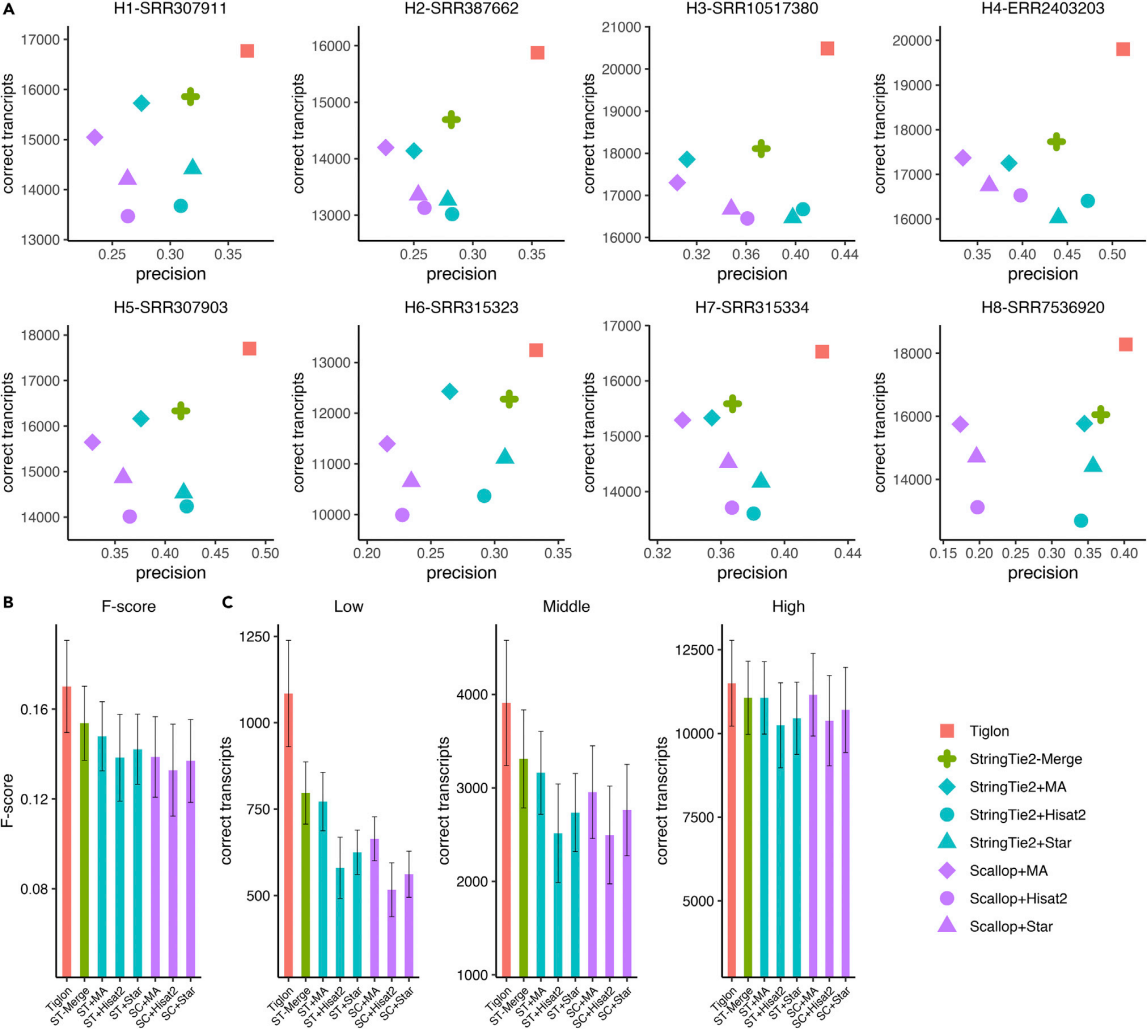
Performance evaluation on the eight *Homo sapiens* samples H1–H8 (A) Precision and the number of correctly assembled transcripts of the assemblers on the eight samples. (B) Average F-score of the assemblers on the eight samples. The error bars show the SD (the same for other panels). (C) The average number of correctly assembled transcripts with different expression levels by the assemblers on the eight samples. The abbreviation ST is for StringTie2, SC for Scallop, and MA for MergedAlignments.

We first mapped the eight *H. sapiens* RNA-seq samples to the reference genome by using Hisat2 and Star, respectively, followed by generating the merged alignments for each sample, and we subsequently ran the assemblers. The results showed that Tiglon reached consistently and significantly a higher number of correctly assembled transcripts and precision than all the other assemblers on all the tested datasets (Figure 3).

Specifically, the correctly recovered transcripts on the eight samples of Tiglon ranged from 13,244 to 20,489, with an average of about 17,377, versus 12,278–18,112 for StringTie2-Merge with an average of 15,831, 12,429–17,857 for StringTie2+MergedAlignments with an average of 15,584, 11,397–17,371 for Scallop + MergedAlignments with an average of 15,251, 10,369–16,671 for StringTie2+Hisat2 with an average of 13,834, 11,116–16,469 for StringTie2+Star with an average of 14,303, 9993–16,531 for Scallop + Hisat2 with an average of 13,802, and 10,653–16,750 for Scallop + Star with an average of 14,470. On the whole, averaged on the eight tested datasets, Tiglon reconstructed 9.51% more correct transcripts than StringTie2-Merge, 11.25% more than StringTie2+MergedAlignments, 13.68% more than Scallop + MergedAlignments, 25.32% more than StringTie2+Hisat2, 21.21% more than StringTie2+Star, 25.61% more than Scallop + Hisat2, and 19.82% more than Scallop + Star (Figure 3A and Tables S3–S10).

The high number of correct transcripts assembled by Tiglon was not at the cost of its precision. In terms of precision, Tiglon still kept the highest on all the tested samples. The average precision of Tiglon on the eight samples reached about 41.27%, while 35.89% for StringTie2-Merge, 32.03% for StringTie2+MergedAlignment, 26.90% for Scallop + MergedAlignments, 36.31% for StringTie2+Hisat2, 36.31% for StringTie2+Star, 30.47% for Scallop + Hisat2, and 29.77% for Scallop + Star. Overall, Tiglon showed an average improvement of 13.67%–53.44% over the other approaches (Figure 3A and Tables S3–S10).

Furthermore, we calculated the F-score for each assembler, and Tiglon remained in its best performance. Averaged on the eight samples, the F-score of Tiglon reached 0.1701, which was about 10.67%–28.08% higher than the other approaches (Figure 3B and Tables S3–S10).

After that, we evaluated the ability of the assemblers in reconstructing transcripts with different expression levels. Although we cannot know the expression abundance for the ground truth, we used the well-known abundance estimator Kallisto (Bray et al., 2016) to quantify the eight RNA-seq samples. Based on the estimated abundance, we classified transcripts into three parts corresponding to low, middle, and high expression levels as we did on the simulated dataset. Comparing among these assemblers, Tiglon consistently achieved the highest number of correctly assembled transcripts on different expression levels upon all the tested samples (Figure 3C and Tables S16–S23). What’s more, Tiglon exhibited a significant superiority over all the others in producing transcripts with low expression levels. On the eight samples, Tiglon correctly recovered 22.57%–53.48% more lowly expressed transcripts than StirngTie2-Merge, 29.73%–62.48% more than StringTie2+MergedAlignments, 40.71%–89.05% more than Scallop + MergedAlignments, 67.65%–135.38% more than StringTie2+Hisat2, 51.93%–87.11% more than StringTie2+Star, 68.20%–161.18% more than Scallop + Hisat2, and 59.28%–116.55% more than Scallop + Star.

#### Performance evaluation on the *M. musculus* samples

**Figure 4:**
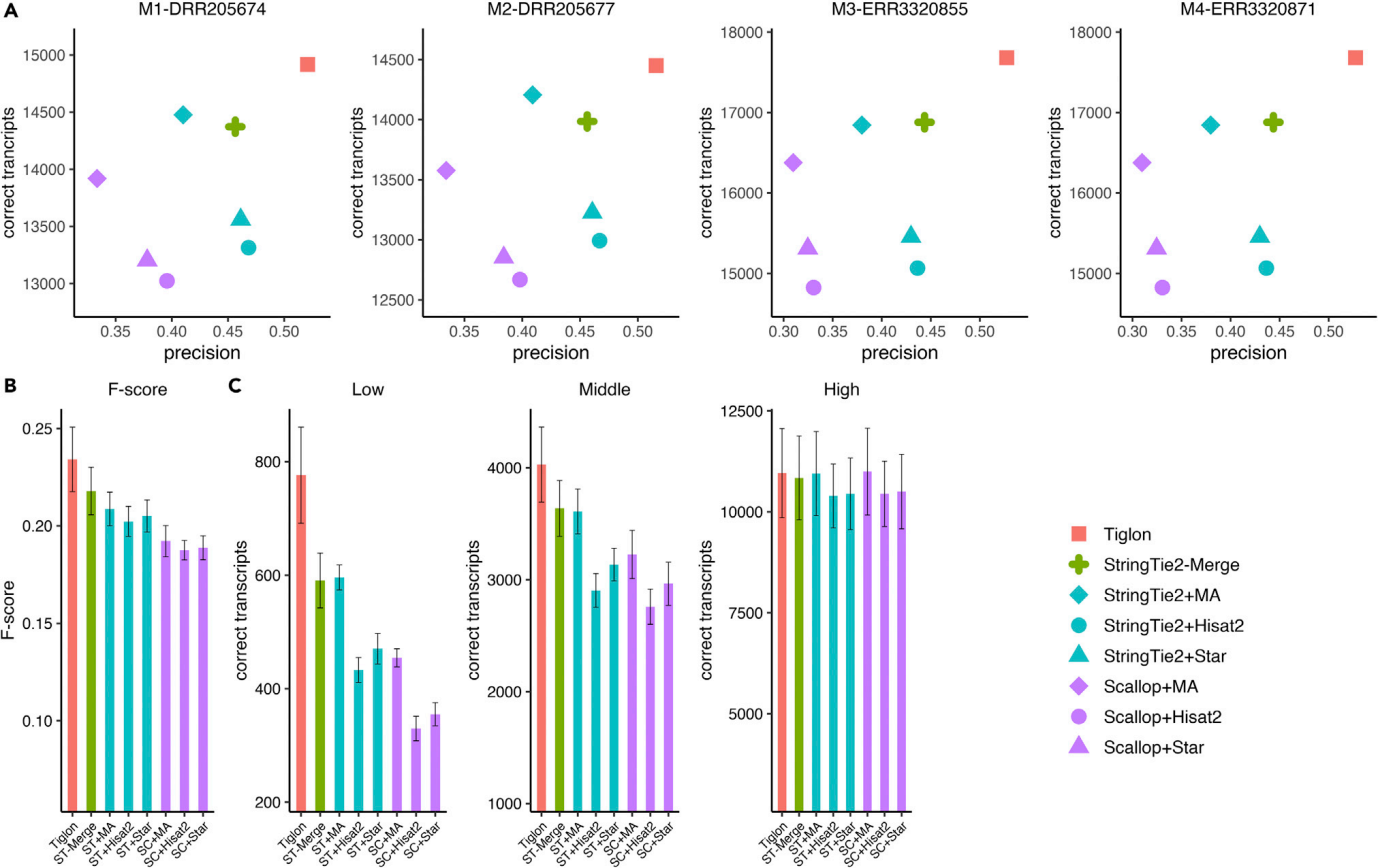
Performance evaluation on the four *Mus musculus* samples M1–M4 (A) Precision and the number of correctly assembled transcripts of the assemblers on the four samples. (B) Average F-score of the assemblers on the four samples. The error bars show the SD (the same for other panels). (C) The average number of correctly assembled transcripts with different expression levels by the assemblers on the four samples. The abbreviation ST is for StringTie2, SC for Scallop, and MA for MergedAlignments.

We then evaluated the performance of Tiglon on the four *M. musculus* samples. We first mapped the four *M. musculus* RNA-seq samples to the reference genome and generated the merged alignments, and then ran the assemblers depending on the alignments. As expected, Tiglon actually demonstrated the best performance (Figure 4).

On the four *M. musculus* samples, the correctly assembled transcripts of Tiglon achieved 14,450–17,683 with an average of 16,153, which was 4.74% higher than StingTie2-Merge, 4.28% higher than StringTie2+MergedAlignments, 14.01% higher than Scallop + MergedAlignments, and 12.72%–17.51% higher than StingTie2 and Scallop depending on aligners. Evaluation in terms of precision, Tiglon again performed better. To be specific, Tiglon reached an average precision of 51.71% with the range from 50.44% to 52.73% on the four samples, versus average precision 44.42% for StringTie2-Merge, 37.75% for StringTie2+MergedAlignments, 31.11% for Scallop + MergedAlignments, 44.31% for StringTie2+Hisat2, 43.64% for StringTie2+Star, 35.27% for Scallop + Hisat2, and 34.16% for Scallop + Star. Overall, Tiglon exhibited an average improvement of 16.40%–66.19%% over the other approaches (Figure 4A and Tables S11–S14).

We also computed the F-score. Averaged on samples, the F-score of Tiglon reached about 0.2341, which was 7.45% higher than StringTie-Merge, 12.19% higher than StringTie2+MergedAlignments, 21.85% higher than Scallop + MergedAlignments, and 14.17%–24.86% higher than StringTie2 and Scallop depending on different aligners (Figure 4B and Tables S11–S14).

As did in the *H. sapiens* samples, we next evaluated the performance of recovering transcripts with different expression levels. The comparison showed that Tiglon outperformed all the others regardless of expression levels on all the four tested samples. Especially in terms of recovering lowly expressed transcripts, Tiglon demonstrated an improvement of 25.36%–39.91% over StringTie2-Merge, 19.65%–45.05% over StringTie2+MergedAlignments, 55.40%–96.11% over Scallop + MergedAlignments, 67.74%–98.69% over StringTie2+Hisat2, 56.12%–79.09% over StringTie2+Star, 126.85%–154.34% over Scallop + Hisat2, and 108.64%–139.58% over Scallop + Star (Figure 4C and Tables S24–S27).

### Performance evaluation on additional samples

With so many public RNA-seq samples available, in addition to the above evaluation, we further ran the assemblers on additional 38 real RNA-seq samples, with 28 from the species of *H. sapiens* and 10 from the *M. musculus* (Table S1 record the accession code and description of these samples). On all these additional tests, Tiglon consistently exhibited the best performance with significant improvements in both the number of correctly assembled transcripts and precision over all the other approaches (Figures S1–S4).

### Performance comparisons between running tiglon with multiple aligners and with only one aligner

Tiglon is specifically designed for integrating multiple alignments to assemble full-length transcripts, where the alignments are generated by different mapping tools. To show the advantages of Tiglon’s integrating technics (here denoted as Tiglon + Hisat2&Star), we further ran Tiglon with the alignments generated by only one aligner (Hisat2 or Star) as its input on the eight *H. sapiens* samples H1–H8, and four *M. musculus* samples M1–M4, denoted as Tiglon + Hisat2 and Tiglon + Star. We compared Tilgon + Hisat2&Star with Tiglon + Hisat2 and Tiglon + Star, and the results demonstrated that Tiglon + Hisat2&Star performed significantly better no matter in terms of precision or the number of correctly assembled transcripts. Averaged on the eight *H. sapiens* samples, Tiglon + Hisat2&Star showed an improvement of 6.47% and 6.91% on precision, and 16.11% and 13.98% on the number of correct transcripts over Tiglon + Hisat2 and Tiglon + Star. And, on the four *M. musculus* samples, the improvements were 9.19% and 7.26%, and 12.17% and 10.05%, respectively (Figures S5–S6 and Tables S3–S14).

### Comparing tiglon with our previous algorithms TransBorrow and iPAC

We then compared Tiglon with our previous algorithms, TransBorrow (Yu et al., 2020b) and iPAC (Yu et al., 2020a), both were single aligner-based assemblers, where TransBorrow that employed a borrowing strategy was run by taking the assemblies of StringTie2 and Scallop as its input, and iPAC was run with its default setting. We evaluated the performance on the eight *H. sapiens* samples H1–H8, and the four *M. musculus* samples M1–M4 under Hisat2 and Star alignments. It is worth mentioning that TransBorrow and iPAC cannot be run with the merged alignments produced by *samtools merge*. The comparing results exhibited that the proposed multiple aligner-based Tiglon algorithm outperformed both TransBorrow and iPAC significantly. For instance, averaged on the eight *H. sapiens* samples, Tiglon correctly recovered 12.2% and 8.5% more transcripts than TransBorrow + Hisat2 and TransBorrow + Star, and 16.1% and 14.0% more than iPAC + Hisat2 and iPAC + Star, while in terms of precision, Tiglon averagely showed an improvement of 4.02%–4.26% over TransBorrow and 6.47%–6.91% over iPAC depending on Hisat2 or Star alignments (see Figures S7, S8 and Tables S3–S14 for details).

### Additional tests

It is worth mentioning that, in this research, we used Hisat2 and Star to produce the alignments for the RNA-seq samples, where Star was run with its default settings, while Hisat2 was run with the option ***--dta*** (in the Hisat2 manual, it means “*reports alignments tailored for transcript assemblers*”). Here, we further used the default settings (without the option ***--dta***) of Hisat2 to generate the alignments. Based on the new produced Hisat2 default alignments and the aforementioned Star alignments, we made the test on the samples H1–H8 and M1–M4 once again, and the testing results exhibited a similar performance trend where Tiglon consistently kept the superior performance (Figure S7).

### Comparison of consumptions of computing resources

All the assemblers were run on the same server with 768 GB of memory and a 32-core CPU. On all the tested datasets, Tiglon ran a little slower than StringTie2 and Scallop, and it costed more memory than StringTie2, which was almost the same as Scallop. For example, on the first dataset H1, which contains 41 million paired-end reads, the running time of StringTie2 was 18 and 17 min based on Hisat2 and Star, respectively, 19 and 19 min for Scallop, and 46 min for Tiglon that ran with the alignments produced by both aligners in parallel. For memory usage, StringTie2 costed the least memory of no more than 1 GB. In contrast, the two assemblers Scallop and Tiglon exhibited a similar trend, with the maximum memory usage of 5–9 GB for Scallop and approximately 8 GB for Tiglon. Overall, Tiglon is not the most economical in running time and memory usage; even so, it is quite acceptable for practical use.

## Discussion

We present Tiglon, a new genome-guided assembler that integrates multiple alignments of different mapping tools to reconstruct transcripts. We mainly focused on the performance comparison between the proposed Tiglon and two of the extremely popular and extensively used assemblers StringTie2 and Scallop, depending on the alignments of Hisat2, Star, and the merged alignments of both. Based on the test results, Tiglon demonstrated a significant superiority in performance on both simulated and real biological datasets. Its advantages may be attributed to 1) integrating alignments of different aligners and building the labeled splice graph capture more splicing junctions than the traditional approaches, which base on only one aligner 2) extracting much more reliable paired paths depending on the labeled splice graph, and 3) the newly developed labeled-based dynamic path-searching techniques for extracting all the transcript-representing paths over the labeled splice graphs. These unique ingredients make the Tiglon algorithm not only highly sensitive but also remarkably precise.

Moreover, we compared Tiglon with two of our previous algorithms TransBorrow and iPAC, where iPAC utilized the phasing graph model, and TransBorrow employed a borrowing strategy (make use of the assembly results of other algorithms). These two assemblers are designed from different angles to generate the assemblies. However, both are not compatible with the merged alignments produced by different aligners. The proposed Tiglon algorithm, which is designed for integrating multiple alignments, exhibits better performance. That is to say, taking multiple alignments into consideration would be a helpful strategy for transcriptome assembly. We hope it would open up new ideas for the researchers to develop better algorithms. From our perspectives, a combination of the proposed integrating strategy and the borrowing strategy that used in the TransBorrow algorithm would be an interesting attempt to improve the assembly accuracy. We will definitely explore this in our future work.

Although the current version of Tiglon exhibits significant advantages, there is still room for further improvements in the future. For instance, the current version is not compatible with long-read RNA-seq datasets (e.g., Pacific Biosciences [PacBio] or Oxford Nanopore Technologies [ONT]). In the future version, we will attempt to solve the problem.

To the best of our knowledge, Tiglon is the first genome-guided transcriptome assembler that is specifically designed to integrate alignments of different mapping tools to build the labeled splice graph and to extract more reliable paired paths. Tiglon employs a dynamic programming algorithm to recover the transcripts by making strategic use of the label information. The software has been developed to be user-friendly. It is expected to play a crucial role in discoveries of transcriptome studies using RNA-seq, especially in complicated human diseases related to abnormal splicing events and expression levels, such as cancers.

### Limitations of the study

We introduce a new tool designed to deliver better performance in transcriptome assembly. The current version of Tiglon does not have an option for annotation-guided assemblies, i.e., where the known transcript annotations are used to guide the assembly procedure. Such annotation-guided assembly is supposed to achieve higher accuracy. We intend in our future work to focus not only on the usage of the sequence alignment but also on the usage of other information, such as the known annotations, and even the assembly results of other tools.

## STAR★Methods

### Key resources table

**Table:** 

REAGENT or RESOURCE	SOURCE	IDENTIFIER
**Deposited data**		

Fastq files for RNA-seq of H1	Sequence Read Archive (SRA) in NCBI	SRA accession: SRR307911
Fastq files for RNA-seq of H2	Sequence Read Archive (SRA) in NCBI	SRA accession: SRR387662
Fastq files for RNA-seq of H3	Sequence Read Archive (SRA) in NCBI	SRA accession: SRR10517380
Fastq files for RNA-seq of H4	Sequence Read Archive (SRA) in NCBI	SRA accession: ERR2403203
Fastq files for RNA-seq of H5	Sequence Read Archive (SRA) in NCBI	SRA accession: SRR307903
Fastq files for RNA-seq of H6	Sequence Read Archive (SRA) in NCBI	SRA accession: SRR315323
Fastq files for RNA-seq of H7	Sequence Read Archive (SRA) in NCBI	SRA accession: SRR315334
Fastq files for RNA-seq of H8	Sequence Read Archive (SRA) in NCBI	SRA accession: SRR7536920
Fastq files for RNA-seq of M1	Sequence Read Archive (SRA) in NCBI	SRA accession: DRR205674
Fastq files for RNA-seq of M2	Sequence Read Archive (SRA) in NCBI	SRA accession: DRR205677
Fastq files for RNA-seq of M3	Sequence Read Archive (SRA) in NCBI	SRA accession: ERR3320855
Fastq files for RNA-seq of M4	Sequence Read Archive (SRA) in NCBI	SRA accession: ERR3320871
Fastq files for RNA-seq of S1	Sequence Read Archive (SRA) in NCBI	SRA accession: SRR545723
Fastq files for RNA-seq of S2	Sequence Read Archive (SRA) in NCBI	SRA accession: SRR534291
Fastq files for RNA-seq of S3	Sequence Read Archive (SRA) in NCBI	SRA accession: SRR8767255
Fastq files for RNA-seq of S4	Sequence Read Archive (SRA) in NCBI	SRA accession: SRR307905
Fastq files for RNA-seq of S5	Sequence Read Archive (SRA) in NCBI	SRA accession: SRR8759122
Fastq files for RNA-seq of S6	Sequence Read Archive (SRA) in NCBI	SRA accession: SRR315326
Fastq files for RNA-seq of S7	Sequence Read Archive (SRA) in NCBI	SRA accession: SRR315330
Fastq files for RNA-seq of S8	Sequence Read Archive (SRA) in NCBI	SRA accession: SRR8867129
Fastq files for RNA-seq of S9	Sequence Read Archive (SRA) in NCBI	SRA accession: SRR8867125
Fastq files for RNA-seq of S10	Sequence Read Archive (SRA) in NCBI	SRA accession: SRR8767256
Fastq files for RNA-seq of S11	Sequence Read Archive (SRA) in NCBI	SRA accession: SRR7478767
Fastq files for RNA-seq of S12	Sequence Read Archive (SRA) in NCBI	SRA accession: SRR7536918
Fastq files for RNA-seq of S13	Sequence Read Archive (SRA) in NCBI	SRA accession: SRR10517375
Fastq files for RNA-seq of S14	Sequence Read Archive (SRA) in NCBI	SRA accession: SRR10517379
Fastq files for RNA-seq of S15	Sequence Read Archive (SRA) in NCBI	SRA accession: SRR10517374
Fastq files for RNA-seq of S16	Sequence Read Archive (SRA) in NCBI	SRA accession: SRR8315697
Fastq files for RNA-seq of S17	Sequence Read Archive (SRA) in NCBI	SRA accession: SRR8315695
Fastq files for RNA-seq of S18	Sequence Read Archive (SRA) in NCBI	SRA accession: SRR7047912
Fastq files for RNA-seq of S19	Sequence Read Archive (SRA) in NCBI	SRA accession: SRR8867128
Fastq files for RNA-seq of S20	Sequence Read Archive (SRA) in NCBI	SRA accession: SRR8588656
Fastq files for RNA-seq of S21	Sequence Read Archive (SRA) in NCBI	SRA accession: SRR10611961
Fastq files for RNA-seq of S22	Sequence Read Archive (SRA) in NCBI	SRA accession: SRR10039475
Fastq files for RNA-seq of S23	Sequence Read Archive (SRA) in NCBI	SRA accession: SRR6013560
Fastq files for RNA-seq of S24	Sequence Read Archive (SRA) in NCBI	SRA accession: ERR3639847
Fastq files for RNA-seq of S25	Sequence Read Archive (SRA) in NCBI	SRA accession: ERR3639846
Fastq files for RNA-seq of S26	Sequence Read Archive (SRA) in NCBI	SRA accession: ERR3639851
Fastq files for RNA-seq of S27	Sequence Read Archive (SRA) in NCBI	SRA accession: ERR3639849
Fastq files for RNA-seq of S28	Sequence Read Archive (SRA) in NCBI	SRA accession: SRR8759124
Fastq files for RNA-seq of S29	Sequence Read Archive (SRA) in NCBI	SRA accession: ERR3502071
Fastq files for RNA-seq of S30	Sequence Read Archive (SRA) in NCBI	SRA accession: SRR11114714
Fastq files for RNA-seq of S31	Sequence Read Archive (SRA) in NCBI	SRA accession: SRR11171673
Fastq files for RNA-seq of S32	Sequence Read Archive (SRA) in NCBI	SRA accession: SRR11171674
Fastq files for RNA-seq of S33	Sequence Read Archive (SRA) in NCBI	SRA accession: DRR205676
Fastq files for RNA-seq of S34	Sequence Read Archive (SRA) in NCBI	SRA accession: DRR205678
Fastq files for RNA-seq of S35	Sequence Read Archive (SRA) in NCBI	SRA accession: ERR3320877
Fastq files for RNA-seq of S36	Sequence Read Archive (SRA) in NCBI	SRA accession: ERR3320873
Fastq files for RNA-seq of S37	Sequence Read Archive (SRA) in NCBI	SRA accession: SRR203276
Fastq files for RNA-seq of S38	Sequence Read Archive (SRA) in NCBI	SRA accession: ERR3320869
Human reference genome, GRCh38/hg38	Genome Reference Consortium	https://hgdownload.soe.ucsc.edu/goldenPath/hg38/chromosomes/
Mouse reference genome, GHCm38/mm10	Genome Reference Consortium	https://hgdownload.soe.ucsc.edu/goldenPath/mm10/chromosomes/
Human reference transcriptome, hg38.ncbiRefSeq.gtf	Genome Reference Consortium	http://genome.ucsc.edu/cgi-bin/hgTables
Mouse reference transcriptome, mm10.ncbiRefSeq.gtf	Genome Reference Consortium	http://genome.ucsc.edu/cgi-bin/hgTables

**Software and algorithms**		

Tiglon	This paper	https://github.com/yutingsdu/Tiglon-v.1.1.git
StringTie2	Kovaka et al. (2019)	https://github.com/gpertea/stringtie/releases/tag/v2.1.4
Scallop	Shao and Kingsford (2017)	https://github.com/Kingsford-Group/scallop/releases/tag/v0.10.2
iPAC	Yu et al. (2020a)	https://sourceforge.net/projects/transassembly/files/
Trans-Borrow	Yu et al. (2020b)	https://sourceforge.net/projects/transcriptomeassembly/files/TransBorrow/
RSEM	Li and Dewey (2011)	http://deweylab.github.io/RSEM/
Hisat2	Kim et al. (2019)	https://github.com/DaehwanKimLab/hisat2/releases/tag/cba6e8cb
Star	Dobin et al. (2013)	https://github.com/alexdobin/STAR/releases/tag/2.5.3a
Samtools	Li et al. (2009)	http://sourceforge.net/projects/samtools/files/samtools/

### Resource availability

#### Lead contact

Further information and requests for resources should be directed to and will be fulfilled by the lead contact, Ting Yu (yutingsdu@163.com).

#### Materials availability

No unique reagents were generated in this study.

### Method details

By combining multiple alignments of different mapping tools, Tiglon constructs a new graph model, namely the labeled splice graph, which integrates the paired-end and sequence depth information generated by different alignments effectively. Based on the labeled splice graph, Tiglon extracts more reliable paired-end paths, and each paired-end path is given a label as well, followed by a label-based dynamic programming path searching strategy to reconstruct the transcripts (See Figure S10 for the flowchart of Tiglon algorithm).

#### Building labeled splice graphs

To make full use of the alignments produced by different mapping tools, we first build the traditional splice graph based on each aligner and then merging them into the so-called the labeled splice graph.

##### Building splice graphs depending on alignments of each aligner in parallel

First, based on alignments of RNA-seq reads to the reference genome, we cluster the reads into different gene loci. The exon-exon splicing junctions are derived from those junction reads. Then for each gene locus, we build the traditional splice graph *G = (V, E)*, in which each node *v* corresponds to an exon and each edge e corresponds to a splicing junction between two exons. Moreover, the edges and nodes are weighted by the number of reads supporting them. It is worth mentioning that we heuristically remove relatively low-weight edges or nodes that may be caused by sequencing errors or unreasonably aligns

After the splice graph is built, the sequence depth information will be projected onto the nodes and edges of the splice graph as their weights. Generally, the weight of each node is defined as the average coverage of the aligned reads to it, and the weight of every edge is defined as the number of spliced reads that span it. Note that if a read is aligned to multiple sites, suppose N, the contribution of this read is recorded as 1/N.

However, there may be quite a number of spurious splicing junctions in the splice graphs caused by sequencing errors or mapping errors. We heuristically remove edges and nodes with relatively low weight via the following criteria. 1) An edge with its weight less than 1 (noting that if a read is aligned to N sites, the contribution of this read is 1/N) then it may correspond to a sequencing or mapping error. 2) If there is a node with several out- (or in-) edges, such that one of them has a much smaller weight than the total out- (or in-) weights (less than 0.1), then it probably represents a spurious splicing junction. 3) If the weight of an out- (or in-) edge is less than 3% of the total in- (or out-) edges, then it is considered as a spurious splicing junction. 4) If the weight of an edge is less than 2% of the average edge weight of the corresponding splice graph, then we will also remove this edge. 5) If the weight of a single node (a node that is without incoming edges and outgoing edges) is less than 10, the node is regarded as a false positive.

In theory, most splicing events in the expressed transcripts can be captured by the edges in the splice graphs, and the sequencing depth information is appropriately used for the graph as the node and edge weights. However, based on the alignments generated by only one aligner, a large number of spurious (or missing) nodes and splicing junctions in the splice graphs may be caused by the mapping errors (As an example in Figure 1, neither Hisat2 nor Star mappings captured the whole junctions of an annotated transcript). Moreover, a large number of paired paths (paths in the graph supported by the paired-end reads) extracted from the traditional splice graph are not reliable. Thus, it is knotty to accurately recover all the expressed transcripts based on the inaccurate splice graph generated using only one aligner. However, via fully integrating the traditional splice graphs produced by different aligners, more accurate splice graphs can be constructed, named the labeled splice graphs.

##### Merging splice graphs of each aligner to generate the labeled splice graph

Suppose that we get *N* aligners to generate the alignments, and for each gene locus, we denote the splice graph generated based on the aligner *i* (i≤N) as *G*_*i*_ = *(E*_*i*_*, V*_*i*_*),* and for each edge $e_{i}\;\in \;E_{i}$, we denote the set of reads that support *e*_*i*_ as *R*_*ei*_. The labeled splice graph *G*_*L*_ *= (E*_*L*_*,V*_*L*_*)* is generated by merging the splice graphs *G*_*i*_ for i∈[1,N], in which the nodes and edges represent all the nodes and edges appearing in *G*_*i*_ *(*i∈[1,N]*)*. Then we label *G*_*L*_ in the following ways, for each edge *e* in *G*_*L*_, if ∃j∈[1,N] such that $e\;\notin \;E_{j}$, then we label *e* with *1*; otherwise, we label it with *2*. In addition, for the edge $e\;\in \;E_{L}$ with label *2*, if there exist *i* and *j* (i,j∈[1,N], and i≠j) such that $R_{ei}\;\neq \;R_{ej}$, we further label it with 2-, otherwise we label it with 2+. Afterwards, the weights of the edges of the labeled splice graph are assigned as the average weights of the edges appearing in each *G*_*i*_ for i≤N*.*

#### Extracting labeled paired paths

To make full use of the paired-end information for guiding more accurate transcript assembly, we extract reliable labeled paired paths from each labeled graph.

First, for each gene locus, we generate a set of paired paths for each aligner *i*, denoted as *PP*_*i*_, which is achieved by the following ways. For each paired-end read *r*_*1*_ and *r*_*2*_, based on the mappings of aligner *i*, if *r*_*1*_ spans a path *p*_*1*_ *= n*_*j1*_*→n*_*j2*_*→ … →n*_*jk*_, *r*_*2*_ spans a path *p*_*2*_ *= n*_*j’1*_*→n*_*j’2*_*→ … →n*_*j’q*_ in graph *G*_*L*_, and *p*_*1*_ and *p*_*2*_ share a compatible sub-path *p*_*in*_ *= n*_*m1*_*→n*_*m2*_*→ … →n*_*ms*_ satisfying *k + q - s ≥ 3*, then the paired path *p* is generated by connecting the two paths *p*_*1*_ and *p*_*2*_ via the shared compatible path *p*_*in*_, where the compatible sub-path means the same sub-path at the right (left) terminal of *p*_*1*_ and the left (right) terminal of *p*_*2*_, and the shared sub-path contains at least one edge of the labeled splice graph (see Figure S11 for an example). After all the paired-end reads mapped to *G*_*L*_ are processed, we obtain a set *PP*_*i*_ of all paired paths depending on the aligner *i*. And it is worth mentioning that different paired-end reads may generate the same paired path. Therefore, for each path $p\;\in \;PP_{i}$, we denote *R*_*pi*_ as the set of paired-end reads to generate *p*, and the number of paired-end reads that generate each paired path is regarded as the coverage of the path, denoted as *cov(p)*.

After generating the paired path set *PP*_*i*_ along with paired-end reads set *R*_*pi*_ of each $p\;\in \;PP_{i}$ for each aligner *i*, we set $PP_{L}\;=\;{\text{U}}_{i\;\in \;\left[1,\;N\right]}PP_{i}$ be the paired path of the labeled splice graph. Then we label each paired path in *PP*_*L*_ as how we label each edge $e\;\in \;E_{L}$ which is described in Merging splice graphs of each aligner to generate the labeled splice graph.

#### Employing a new label-based dynamic programming path extraction algorithm

Theoretically, the expressed transcripts of a gene correspond to a path cover in the labeled splice graph. Moreover, each labeled paired path corresponds to a segment of an expressed transcript and should be covered by at least one predicted transcript. To achieve this goal, we strategically use the label information and the reliable labeled paired paths in the labeled splice graph and employ a new labeled-based dynamic programming algorithm that is similar to our previous study (Yu et al., 2021) to generate the transcript-representing path cover over each labeled graph. In detail, we recover the expressed transcripts by the following steps.

##### Step1. Choosing a seed and generate a subgraph from the seed

A seed is an edge or a paired path in the labeled splice graph that can further grow into a full-length transcript-representing path. Choose an unused paired path of label 2+ with the largest weight as the seed. If such kinds of paired paths do not exist or they have been all included in the assembled transcripts, the seed will be chosen in the following order: paired paths with label 2-, edges with label 2+, edges with label 2-, paired paths with label 1 or edges with label 1. Paired paths or edges with label 2 mean that they are captured by multiple aligners. That is, they are more likely to be true positives, and such seeds will grow into expressed full-length transcripts with higher probability.

Suppose the chosen seed be *S = v*_*k*_*→v*_*k+1*_*→ … →v*_*l*_ for a paired path or *S = v*_*k*_*→v*_*l*_ for an edge. We extend it to all the right (left) neighbors of node *v*_*k*_ *(v*_*l*_*)* and keep extending until all the neighbors encounter a node without out- (in-) edges, then a subgraph *G*_*S*_ of *G*_*L*_ is generated from the chosen seed.

##### Step2. Extending paths via label-based dynamic programming

We expect to find the most highly expressed transcripts from the extended subgraph *G*_*S*_, which is achieved by using a dynamic programming algorithm. The idea behind the dynamic programming algorithm is to progressively discover the path of higher weight from the seed to every other edge.

We first add an origin (destination) node to the subgraph *G*_*S*_ by connecting it to all the nodes without in- (out-) edges, and set the weights and labels of the newly added edges to be ∞ and 1, respectively. Suppose that the edges in *G*_*S*_ are ordered by topological sort. Without loss of generality, we number them as *0*, *1, 2 … N*. For each edge *i = v→w* in *G*_*S*_, where *v* and *w* are the endpoints of edge *i*, we denote *In_edges(i)* as the set of in-coming edges of node *v, Out_edges(i)* the set of out-going edges of node *w,* and *weight(i)* the coverage of edge *i.*

Assume that the edge *v*_*k*_*→v*_*k+1*_ in the seed *S* (note that *S= v*_*k*_*→v*_*k+1*_*→ … →v*_*l*_ for a paired path seed or *S = v*_*k*_*→v*_*l*_ for an edge seed) corresponds to edge *m* and the edge *v*_*l-1*_*→v*_*l*_ in the seed *S* corresponds to edge *n* (note that if the seed is an edge then *m = n,* otherwise *m<n*).

The algorithm maintains two variables *Exp*_*i*_ and *Pre*_*i*_ *(Suc*_*i*_*)* for edge *i* from *n* to *N* (from *m* to *0*), where *Exp*_*i*_ represents an upper bound on the highest expression of the path from the seed to edge *i* and *Pre*_*i*_ *(Suc*_*i*_*)* is the predecessor (successor) edge of *i*. The dynamic programming algorithm starts with *Exp*_*m*_ *= cov(S), Exp*_*n*_ *= cov(S), Exp*_*i*_ *=* ∞ (*i<m* or *i>n*)*,* and *Pre*_*i*_ = *null* for *i>=n, Suc*_*i*_ *= null* for *i<=m.*

For *i* from *n+1* to *N*, choose an edge *i’* of label 2+ with the largest *Exp*_*i’*_ from *In_edges(i)* (if there doesn’t exist edges with label 2+, then choose the edge *i’* in the following order: edges with label 2-, edges with label 1)*.* And then set *Exp*_*i*_ to be *min{weight(i), Exp*_*i’*_*}* and *Pre*_*i*_ to be *i’.* For *i* from *m-1* to *1,* we use the same way to set *Exp*_*i*_ and *Suc*_*i*_.

Starting at the edge of the largest *Exp* linked with the destination (origin) and extending backward (forward) based on the tag *Pre (Suc)* until reaching the seed *S*, the highest expressed transcript *p*_*h*_ in graph *G*_*S*_ is recovered (see Figure S12 for an example).

##### Step3. Updating labeled splice graph and repeating the procedure

Defining *f*_*min*_ as the minimum edge weight in the extended path *p*_*h*_, we update the weight *weight(e)* to be *weight(e)-f*_*min*_ for each edge *e* in *p*_*h*_, and if *weight(e)* is equal to 0 after updating, we’ll set its label to be 1.

Repeat the path extraction procedure Step1 to Step 3, until all the edges in graph *G*_*L*_ have been covered by the predicted transcripts. Finally, a transcript-representing path cover is obtained, where all the paired paths have been covered by the assembled transcripts.

## Data Availability

Tiglon is implemented in C++ and is freely available as open-source software at https://github.com/yutingsdu/Tiglon-v.1.1.git. The simulated data set can be downloaded from https://sourceforge.net/projects/tiglon/files/SimulatedDataset/. The assembled results of each assembler were also uploaded to website https://sourceforge.net/projects/tiglon/files/Datasets/. The reference genome of the *Homo sapiens* (version: GRCh38/hg38) and *Mus musculus* (version: GRCm38/mm10) were downloaded from the UCSC Genome Browser at https://hgdownload.soe.ucsc.edu/goldenPath/hg38/chromosomes/ and https://hgdownload.soe.ucsc.edu/goldenPath/mm10/chromosomes/, respectively. And the transcriptome (NCBI RefSeq in GTF format) of the species *Homo sapiens* and *Mus musculus* were downloaded from http://genome.ucsc.edu/cgi-bin/hgTables. And the accession code and description of all the RNA-seq samples used in this study were detailed in Table S1. Besides, the running command for each tool can be found in a shell script from https://sourceforge.net/projects/tiglon/files. Any additional information required to reanalyze the data reported in this article is available upon request from the primary contact.
