# Supplementary material for: Tiglon enables accurate transcriptome assembly via integrating mappings of different aligners
Source: iScience. 2022 Mar 12;25(4):104067. doi: 10.1016/j.isci.2022.104067 (PMC8958329; doi:10.1016/j.isci.2022.104067)
Supplement: Document S1. Figures S1–S1 — 2 and Tables S1–27 [file mmc1.pdf]

iScience, Volume *25*

Supplemental information

Tiglon enables accurate transcriptome assembly via integrating mappings of different aligners

Xiaoyu Zhao and Ting Yu

1. Supplemental Figures

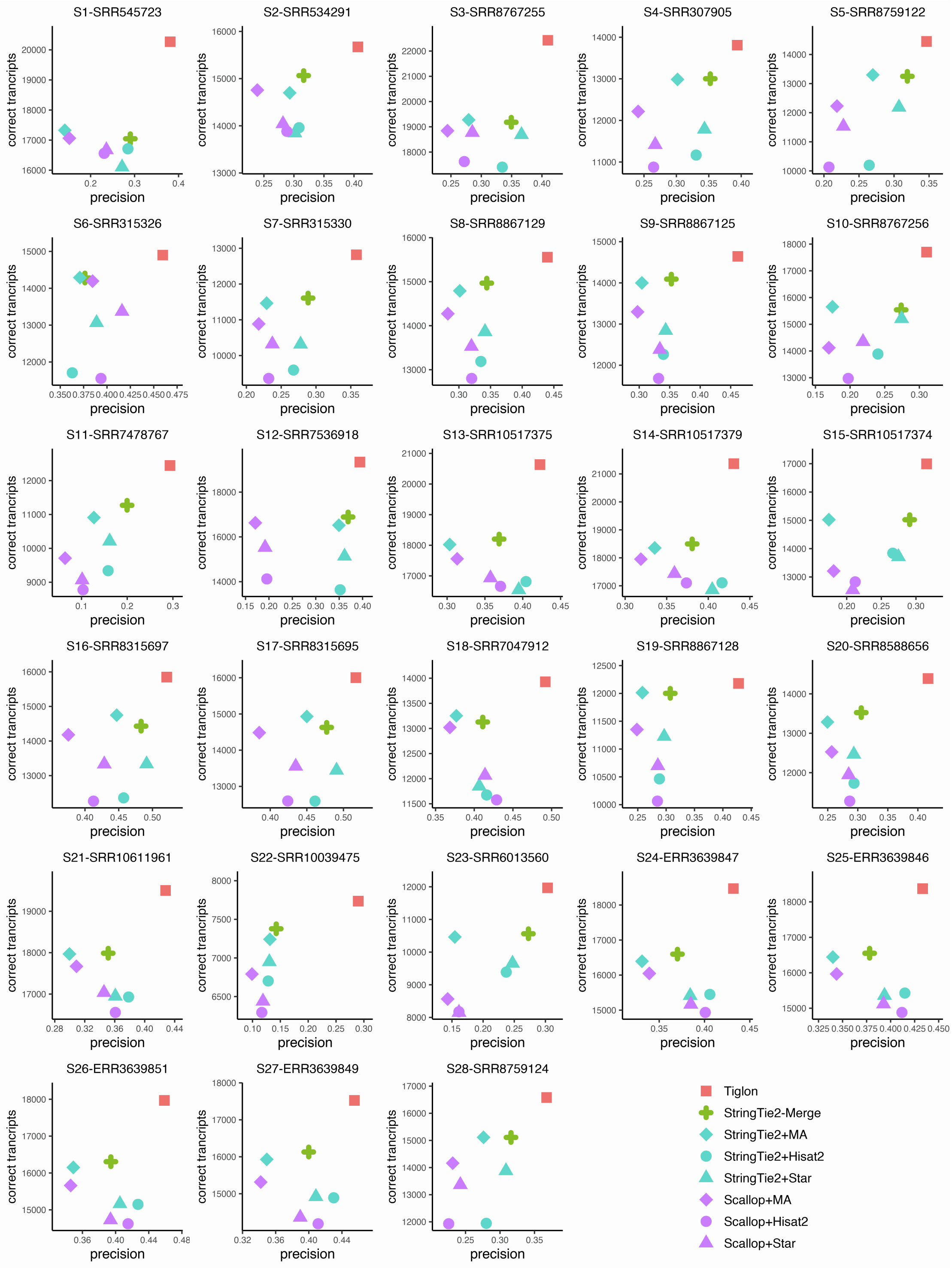

Figure S1 Precision and the number of correctly assembled transcripts of assemblers on the additional 28 Homo sapiens samples S1-S28. The abbreviation MA is for MergedAlignments. Related to Figure 3.

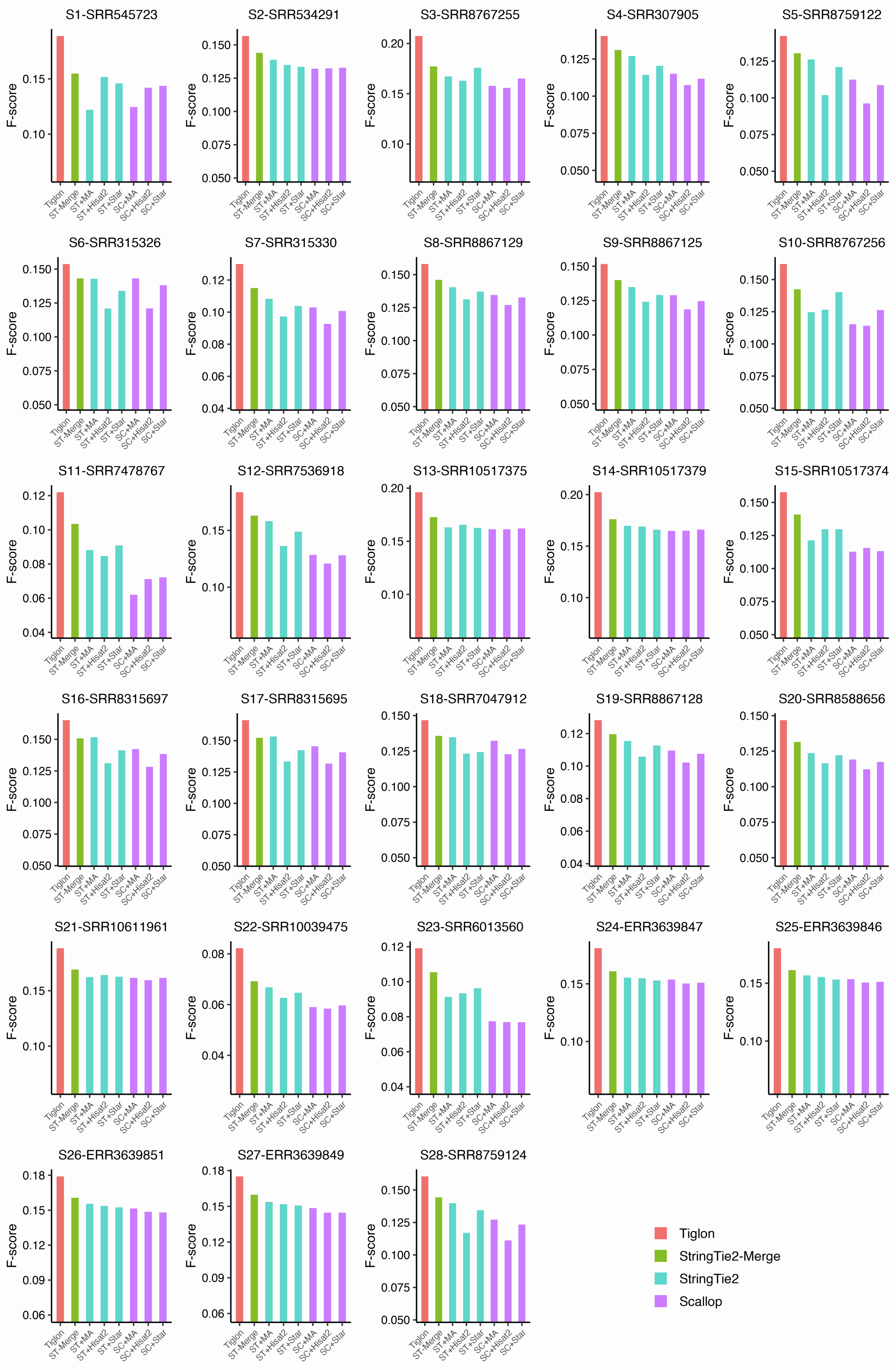

Figure S2 F-score of assemblers on the additional 28 Homo sapiens samples S1-S28. The abbreviation ST is for StringTie2, SC for Scallop, and MA for MergedAlignments. Related to Figure 3.

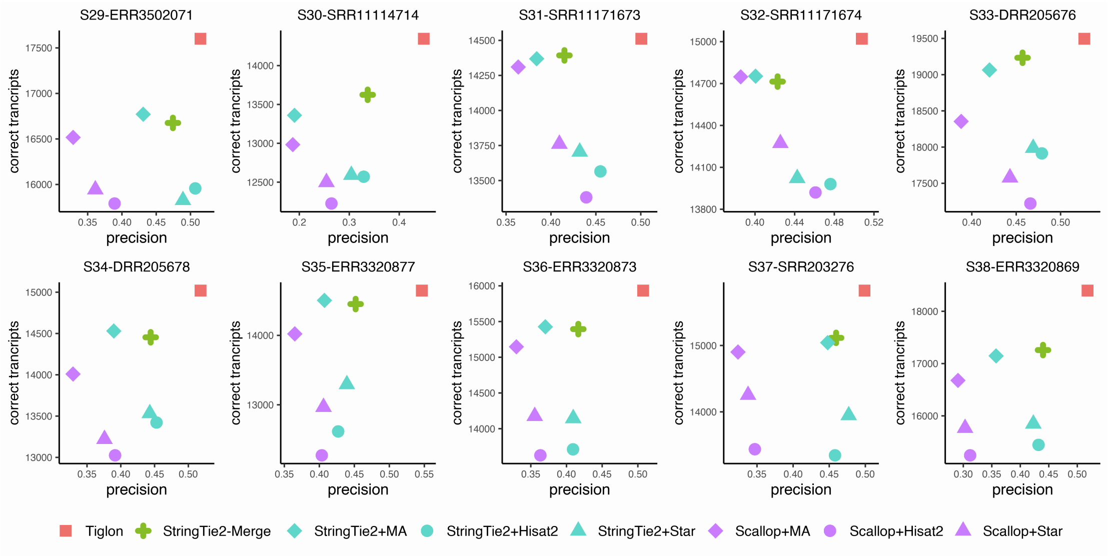

Figure S3 Precision and the number of correctly assembled transcripts of assemblers on the additional 10 Mus musculus samples S29-S38. The abbreviation MA is for MergedAlignments. Related to Figure 4.

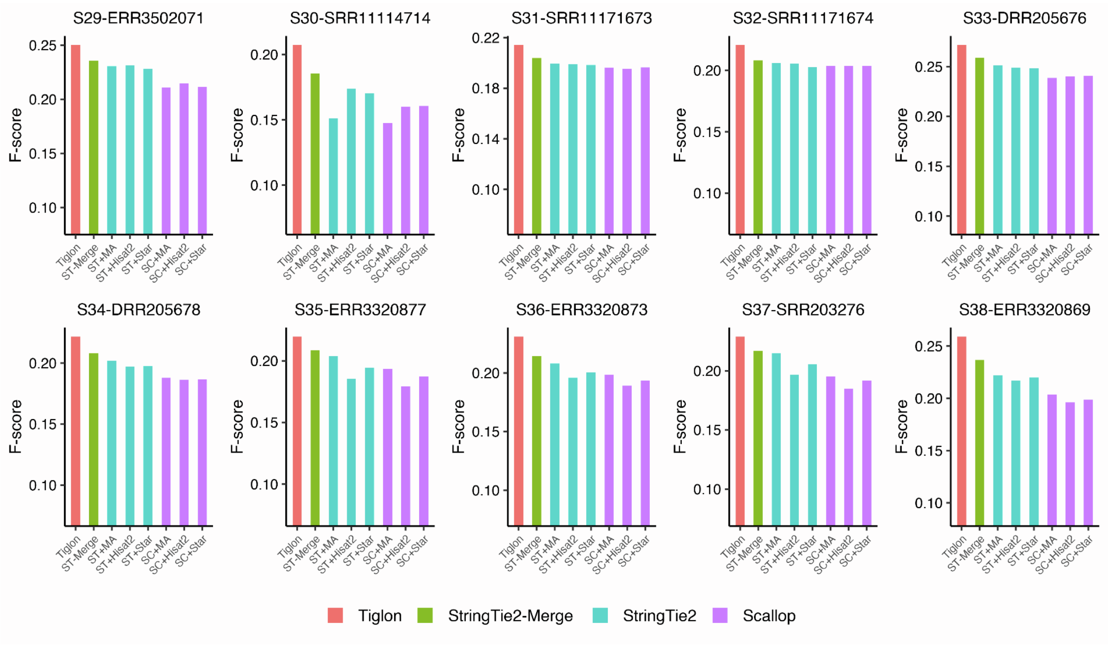

Figure S4 F-score of assemblers on the additional 10 Mus musculus samples S29-S38. The abbreviation ST is for StringTie2, SC for Scallop, and MA for MergedAlignments. Related to Figure 4.

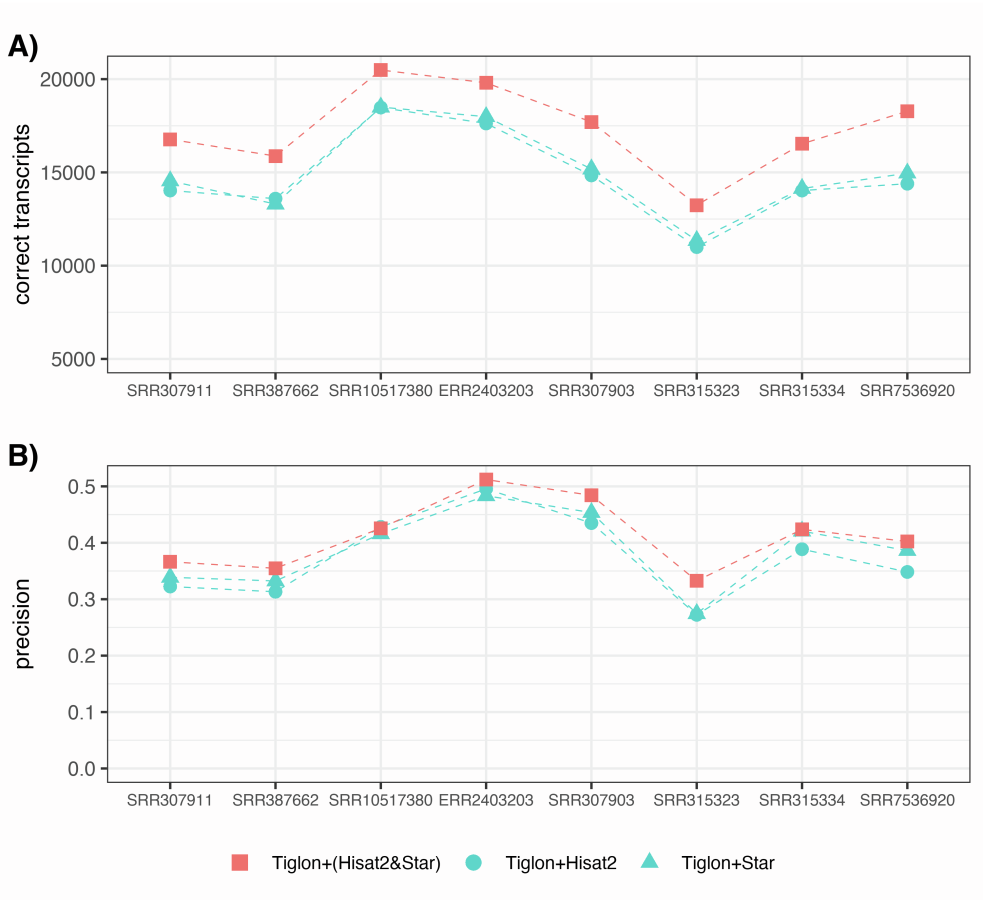

Figure S5. Comparisons between running Tiglon with two aligners (Hisat2 and Star) and only one aligner (Hisat2 or Star) on the eight Homo sapiens samples H1-H8. A) Comparisons of the number of correctly assembled transcripts. B) Comparisons of precision. Related to Figure 3.

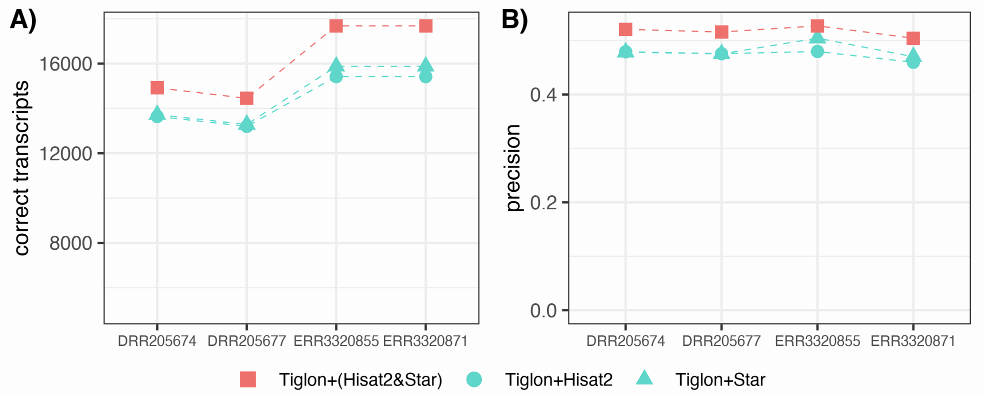

Figure S6 Comparisons between running Tiglon with two aligners (Hisat2 and Star) and only one aligner (Hisat2 or Star) on the four Mus musculus samples M1-M4. A) Comparisons of the number of correctly assembled transcripts. B) Comparisons of precision. Related to Figure 4.

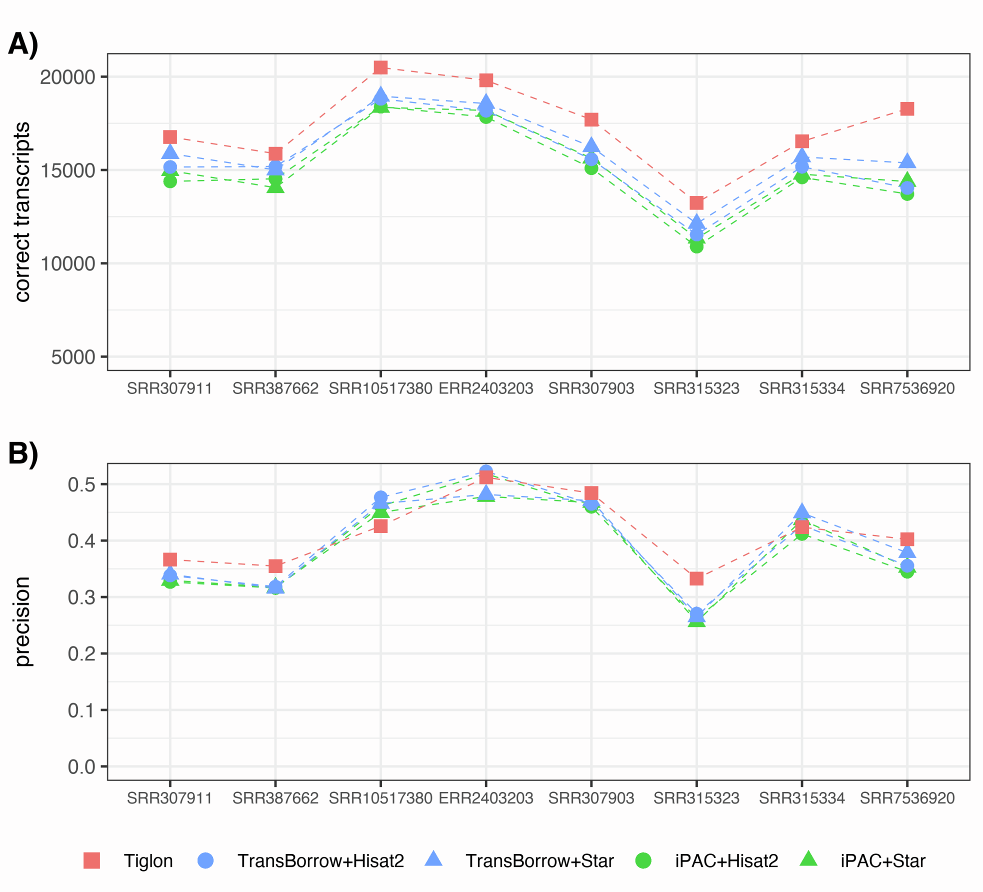

Figure S7. Comparisons between Tiglon and two of our previous algorithms TransBorrow and iPAC on the eight Homo sapiens samples H1-H8. A) Comparisons of the number of correctly assembled transcripts. B) Comparisons of precision. Related to Figure 3.

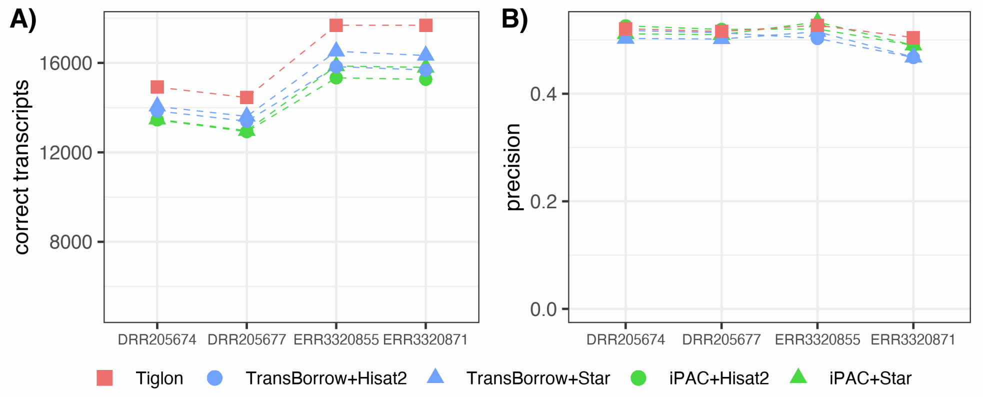

Figure S8 Comparisons between Tiglon and two of our previous algorithms TransBorrow and iPAC on the four Mus musculus samples M1-M4. A) Comparisons of the number of correctly assembled transcripts. B) Comparisons of precision. Related to Figure 4.

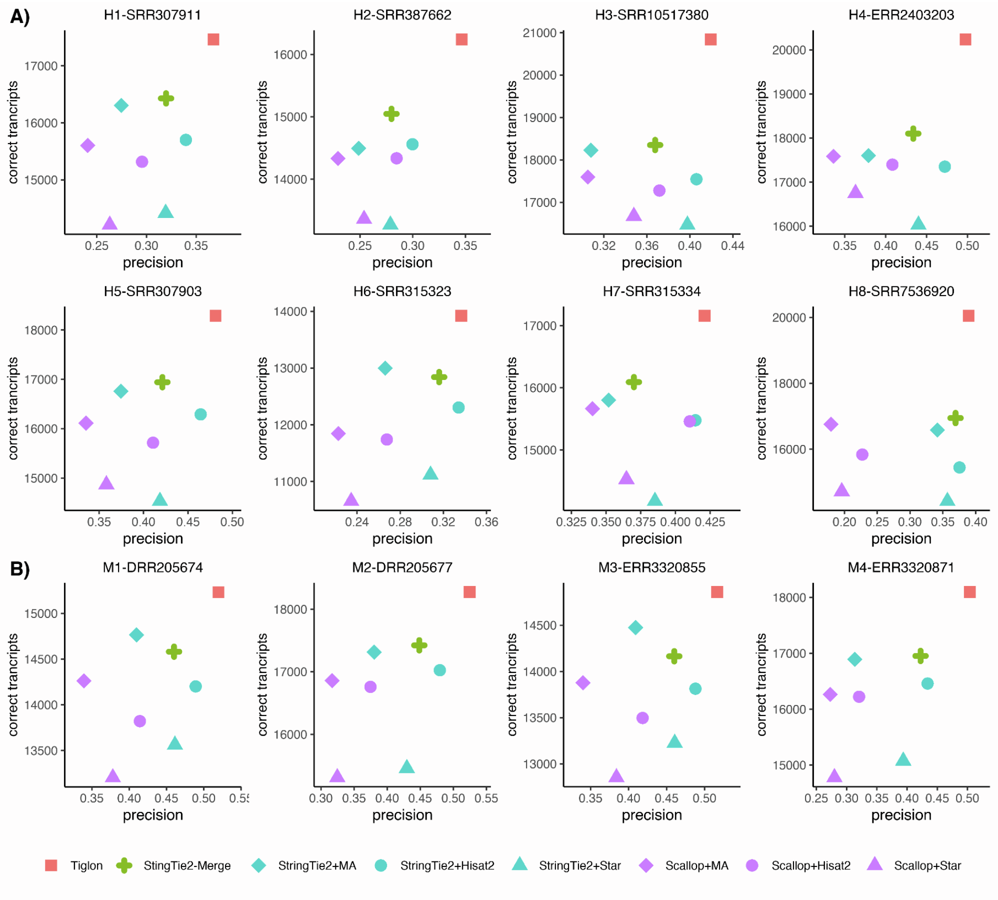

Figure S9. Performance evaluation where Hisat2 was run by using the default settings (without the option ***--dta***). **A)** Precision and the number of correctly assembled transcripts on the Homo sapiens samples H1-H8. **B)** Precision and the number of correctly assembled transcripts on the Mus musculus samples M1-M4. The abbreviation MA is for MergedAlignments. Related to Figure 3.

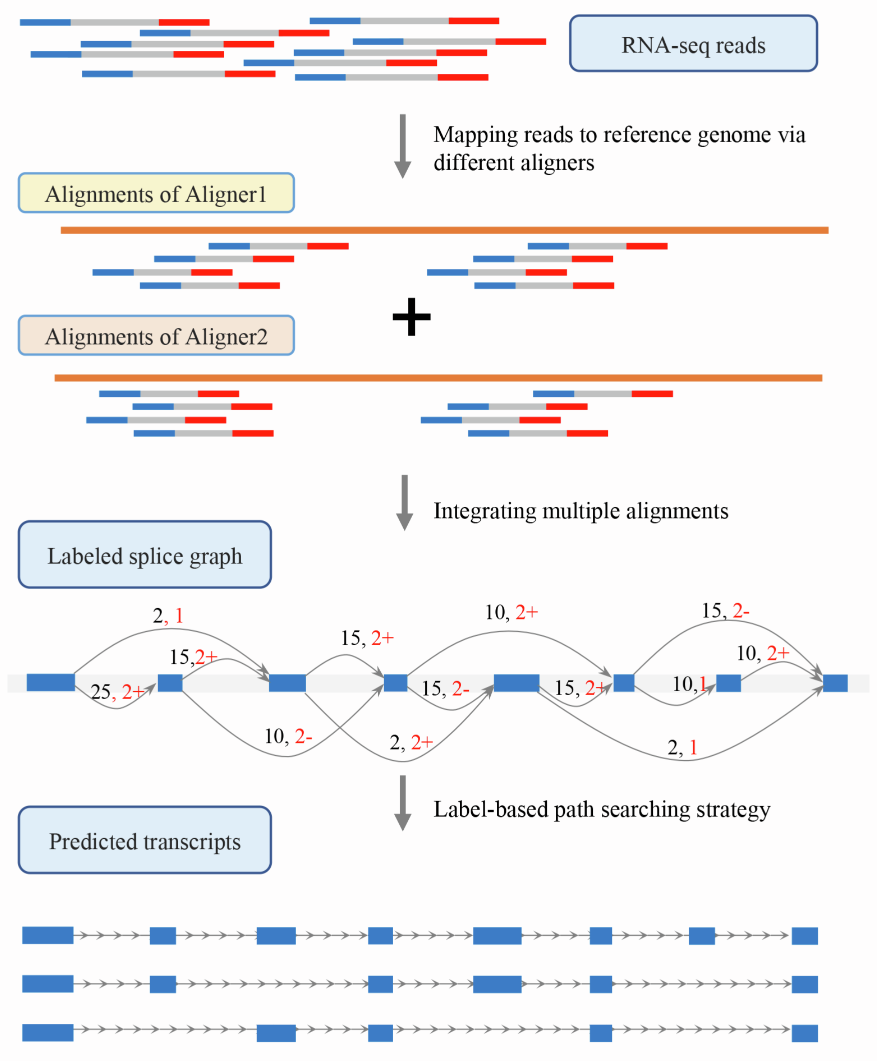

Figure S10. Flowchart of Tiglon algorithm. Related to STAR Methods.

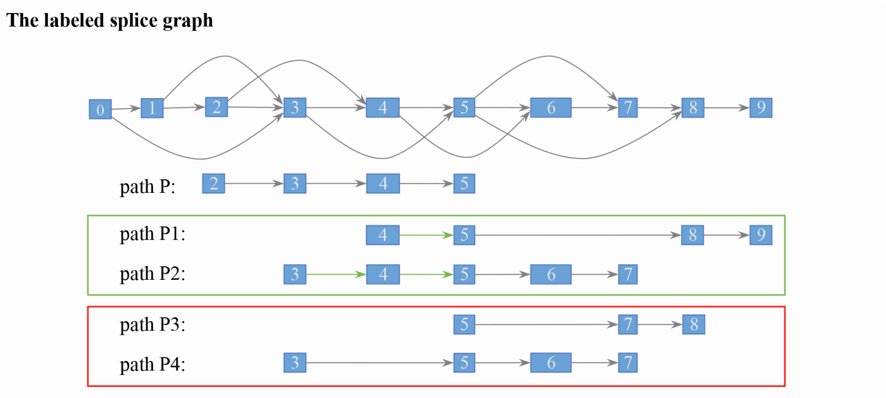

Figure S11. An example illustrating the compatible sub-path between paths. Based on the definition of the compatible sub-path, paths P and P1 possess a compatible sub-path 4-5, to generate the paired path 2-3-4-5-8-9; P and P2 possess a compatible sub-path 3-4-5, to generate the paired path 2-3-4-5-6-7; while P3 and P4 have no compatible sub-path with P. Related to STAR Methods.

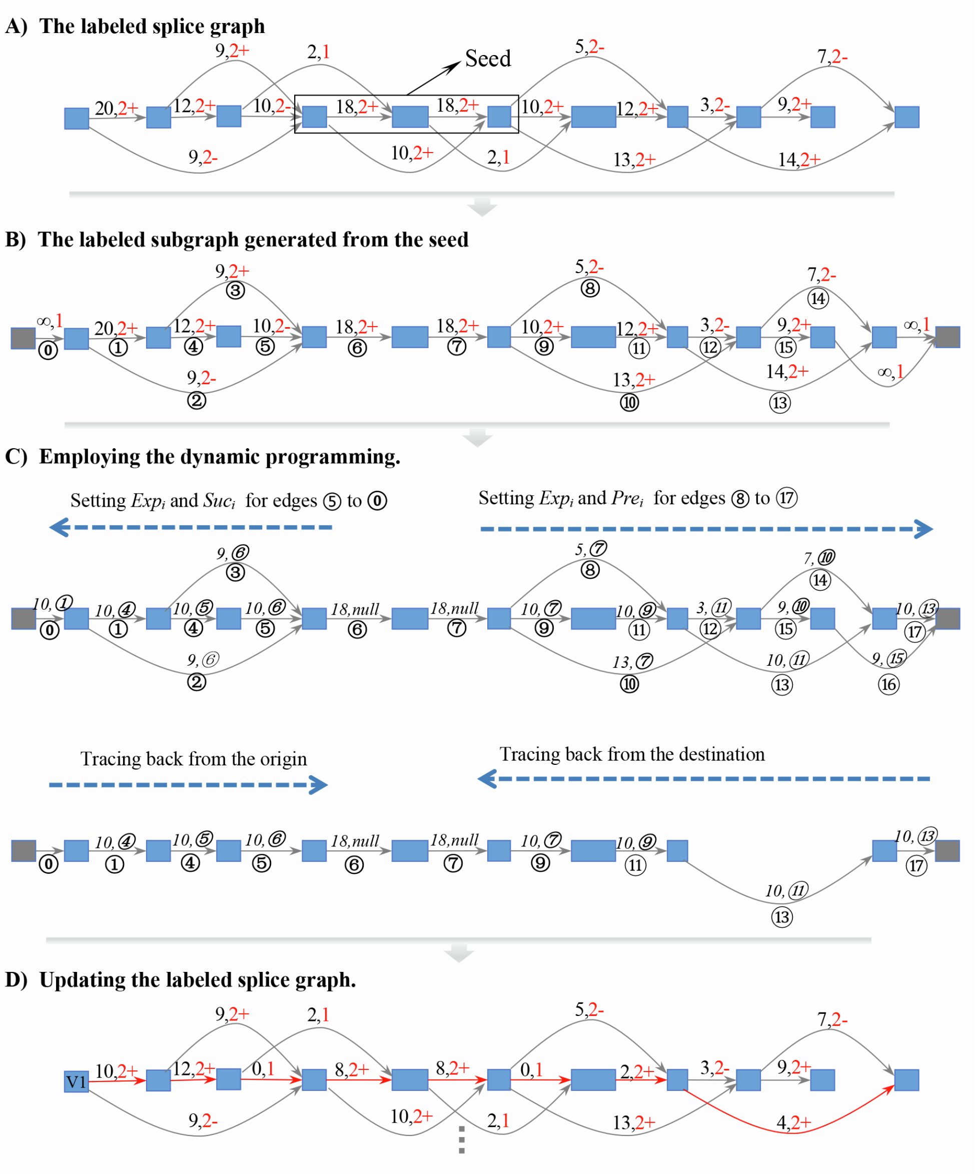

Figure S12. Path extension in a labeled splice graph. A) Linearly arranging the nodes of a labeled splice graph by a topological sort. The number in black on each edge represents the weight of the edge, and the number in red is the label. B) Generating the subgraph from a given seed. The circled number under each edge represents the number of the edge in the subgraph. C) Setting the variables Exp_i and Pre_i (Suc_i) from the seed to every other edge, and extracting the most highly expressed transcript by a traceback loop based on the variables Pre_i (Suc_i). D) Updating the labeled splice graph by subtracting the expression abundance of the extracted transcript from all of the edges contained in the transcript. Related to STAR Methods.

2. Supplemental Tables

Table S1 Summary of the RNA-seq data sets used in this study. Related to STAR Methods.

	SRA Accession	# of Spots	Read length	Species	Layout
H1	SRR307911	41,419,823	76bp	Homo sapiens	paired
H2	SRR387662	88,445,339	76bp	Homo sapiens	paired
H3	SRR10517380	30,715,016	101bp	Homo sapiens	paired
H4	ERR2403203	43,012,995	100bp	Homo sapiens	paired
H5	SRR307903	36,010,036	76bp	Homo sapiens	paired
H6	SRR315323	30,268,824	76bp	Homo sapiens	paired
H7	SRR315334	39,455,691	76bp	Homo sapiens	paired
H8	SRR7536920	52,953,348	50bp	Homo sapiens	paired
M1	DRR205674	25,948,210	101bp	Mus musculus	paired
M2	DRR205677	23,318,089	101bp	Mus musculus	paired
M3	ERR3320855	37,147,357	76bp	Mus musculus	paired
M4	ERR3320871	45,366,428	76bp	Mus musculus	paired
S1	SRR545723	146,844,047	101bp	Homo sapiens	paired
S2	SRR534291	114,006,059	101bp	Homo sapiens	paired
S3	SRR8767255	65,787,471	100bp	Homo sapiens	paired
S4	SRR307905	28,595,307	76bp	Homo sapiens	paired
S5	SRR8759122	41,007,230	52bp	Homo sapiens	paired
S6	SRR315326	36,490,394	76bp	Homo sapiens	paired
S7	SRR315330	36,936,850	76bp	Homo sapiens	paired
S8	SRR8867129	46,900,881	76bp	Homo sapiens	paired
S9	SRR8867125	27,114,142	76bp	Homo sapiens	paired
S10	SRR8767256	53,112,164	100bp	Homo sapiens	paired
S11	SRR7478767	48,854,189	75bp	Homo sapiens	paired
S12	SRR7536918	61,994,219	50bp	Homo sapiens	paired
S13	SRR10517375	30,972,131	101bp	Homo sapiens	paired
S14	SRR10517379	32,795,723	101bp	Homo sapiens	paired
S15	SRR10517374	34,346,963	101bp	Homo sapiens	paired
S16	SRR8315697	13,920,787	76bp	Homo sapiens	paired
S17	SRR8315695	15,938,350	76bp	Homo sapiens	paired

S18	SRR7047912	12,528,527	100bp	Homo sapiens	paired
S19	SRR8867128	34,658,407	76bp	Homo sapiens	paired
S20	SRR8588656	35,346,464	75bp	Homo sapiens	paired
S21	SRR10611961	23,484,713	146bp	Homo sapiens	paired
S22	SRR10039475	29,687,634	125bp	Homo sapiens	paired
S23	SRR6013560	23,290,279	100bp	Homo sapiens	paired
S24	ERR3639847	35,056,571	134bp	Homo sapiens	single
S25	ERR3639846	32,180,589	134bp	Homo sapiens	single
S26	ERR3639851	29,681,809	134bp	Homo sapiens	single
S27	ERR3639849	29,167,280	131bp	Homo sapiens	single
S28	SRR8759124	63,276,904	52bp	Homo sapiens	paired
S29	ERR3502071	27,463,997	150bp	Mus musculus	paired
S30	SRR11114714	44,758,212	151bp	Mus musculus	paired
S31	SRR11171673	18,425,690	150bp	Mus musculus	paired
S32	SRR11171674	19,193,369	150bp	Mus musculus	paired
S33	DRR205676	28,028,951	101bp	Mus musculus	paired
S34	DRR205678	27,327,190	101bp	Mus musculus	paired
S35	ERR3320877	20,066,417	76bp	Mus musculus	paired
S36	ERR3320873	46,740,596	76bp	Mus musculus	paired
S37	SRR203276	52,645,238	76bp	Mus musculus	paired
S38	ERR3320869	43,694,729	76bp	Mus musculus	paired

Table S2. Correct transcripts, assembled transcripts, precision, recall and F-score of the assemblers on simulated dataset. Related to Figure 2.

Assemblers	Correct transcripts	Assembled transcripts	Precision	Recall	F-score
Tiglon+(Hisat2&Star)	22928	35817	0.6401	0.3836	0.4797
Tiglon+Hisat2	20834	34083	0.6113	0.3485	0.4439
Tiglon+Star	20889	33049	0.6321	0.3495	0.4501
StringTie-Merge	20630	36575	0.5640	0.3451	0.4282
StringTie2+MergedAlignm	20654	36320	0.5687	0.3455	0.4299
StringTie2+Hisat2	18800	31897	0.5894	0.3145	0.4102
StringTie2+Star	19267	32758	0.5882	0.3223	0.4164
Scallop+MergedAlignment	20277	55090	0.3681	0.3392	0.3531
Scallop+Hisat2	18786	35542	0.5286	0.3143	0.3942
Scallop+Star	19463	46790	0.4160	0.3256	0.3653
iPAC+Hisat2	19650	32672	0.6014	0.3287	0.4251
iPAC+Star	19330	32179	0.6007	0.3234	0.4204
TransBorrow+Hisat2	19695	31047	0.6344	0.3295	0.4337
TransBorrow+Star	20003	31659	0.6318	0.3346	0.4375

Table S3. Correct transcripts, assembled transcripts, precision, recall and F-score of the assemblers on the Homo sapiens samples H1 (SRR307911). Related to Figure 3.

Assemblers	Correct transcript	Assembled transcripts	Precision	Recall	F-score
Tiglon+(Hisat2&Star)	16772	45783	0.3663	0.1038	0.1618
Tiglon+Hisat2	14029	43505	0.3225	0.0869	0.1369
Tiglon+Star	14541	42913	0.3388	0.0900	0.1423
StringTie-Merge	15859	49954	0.3175	0.0982	0.1500
StringTie2+MergedAlignmentsnts	15729	57153	0.2752	0.0974	0.1439
StringTie2+Hisat2	13675	44241	0.3091	0.0847	0.1329
StringTie2+Star	14418	45149	0.3193	0.0893	0.1395
Scallop+MergedAlignments	15047	64052	0.2349	0.0932	0.1334
Scallop+Hisat2	13470	51130	0.2634	0.0834	0.1267
Scallop+Star	14209	54004	0.2631	0.0880	0.1319
iPAC+Hisat2	14390	44046	0.3267	0.0891	0.1400
iPAC+Star	14963	45393	0.3296	0.0926	0.1446
TransBorrow+Hisat2	15159	44827	0.3382	0.0939	0.1469
TransBorrow+Star	15884	46674	0.3403	0.0983	0.1526

Table S4. Correct transcripts, assembled transcripts, precision, recall and F-score of the assemblers on the Homo sapiens samples H2 (SRR387662). Related to Figure 3.

Assemblers	Correct transcript	Assembled transcripts	Precision	Recall	F-score
Tiglon+(Hisat2&Star)	15876	44748	0.3548	0.0983	0.1539
Tiglon+Hisat2	13593	43403	0.3132	0.0842	0.1327
Tiglon+Star	13302	40026	0.3323	0.0824	0.1320
StringTie-Merge	14693	52144	0.2818	0.0910	0.1375
StringTie2+MergedAlignmentsnts	14140	56528	0.2501	0.0875	0.1297
StringTie2+Hisat2	13019	46092	0.2825	0.0806	0.1254
StringTie2+Star	13267	47583	0.2788	0.0821	0.1269
Scallop+MergedAlignments	14198	62753	0.2263	0.0879	0.1266
Scallop+Hisat2	13130	50690	0.2590	0.0813	0.1237
Scallop+Star	13359	52628	0.2538	0.0827	0.1248
iPAC+Hisat2	14530	45974	0.3160	0.0900	0.1401
iPAC+Star	14053	44307	0.3172	0.0870	0.1366
TransBorrow+Hisat2	15186	47698	0.3184	0.0940	0.1452
TransBorrow+Star	15003	47533	0.3156	0.0929	0.1435

Table S5. Correct transcripts, assembled transcripts, precision, recall and F-score of the assemblers on the Homo sapiens samples H3 (SRR10517380). Related to Figure 3.

Assemblers	Correct transcript	Assembled transcripts	Precision	Recall	F-score
Tiglon+(Hisat2&Star)	20489	48123	0.4258	0.1269	0.1955
Tiglon+Hisat2	18476	43150	0.4282	0.1144	0.1805
Tiglon+Star	18501	44484	0.4159	0.1145	0.1796
StringTie-Merge	18112	48658	0.3722	0.1121	0.1723
StringTie2+MergedAlignmentsnts	17857	57213	0.3121	0.1106	0.1633
StringTie2+Hisat2	16671	41060	0.4060	0.1032	0.1646
StringTie2+Star	16469	41414	0.3977	0.1020	0.1623
Scallop+MergedAlignments	17303	56862	0.3043	0.1071	0.1585
Scallop+Hisat2	16453	45578	0.3610	0.1019	0.1589
Scallop+Star	16677	47927	0.3480	0.1033	0.1592
iPAC+Hisat2	18391	39882	0.4611	0.1139	0.1826
iPAC+Star	18355	40804	0.4498	0.1136	0.1814
TransBorrow+Hisat2	18814	39479	0.4766	0.1165	0.1872
TransBorrow+Star	18953	40690	0.4658	0.1173	0.1875

Table S6. Correct transcripts, assembled transcripts, precision, recall and F-score of the assemblers on the Homo sapiens samples H4 (ERR2403203). Related to Figure 3.

Assemblers	Correct transcript	Assembled transcripts	Precision	Recall	F-score
Tiglon+(Hisat2&Star)	19802	38671	0.5121	0.1226	0.1978
Tiglon+Hisat2	17624	35563	0.4956	0.1091	0.1788
Tiglon+Star	17986	37209	0.4834	0.1114	0.1810
StringTie-Merge	17732	40495	0.4379	0.1098	0.1756
StringTie2+MergedAlignmentsnts	17253	44806	0.3851	0.1068	0.1672
StringTie2+Hisat2	16406	34705	0.4727	0.1016	0.1672
StringTie2+Star	16031	36435	0.4400	0.0993	0.1620
Scallop+MergedAlignments	17371	52042	0.3338	0.1075	0.1627
Scallop+Hisat2	16531	41540	0.3980	0.1023	0.1628
Scallop+Star	16750	46132	0.3631	0.1037	0.1613
iPAC+Hisat2	17837	34410	0.5184	0.1104	0.1821
iPAC+Star	18192	38037	0.4783	0.1126	0.1823
TransBorrow+Hisat2	18176	34788	0.5225	0.1125	0.1852
TransBorrow+Star	18562	38533	0.4817	0.1149	0.1856

Table S7. Correct transcripts, assembled transcripts, precision, recall and F-score of the assemblers on the Homo sapiens samples H5 (SRR307903). Related to Figure 3.

Assemblers	Correct transcript	Assembled transcripts	Precision	Recall	F-score
Tiglon+(Hisat2&Star)	17703	36576	0.4840	0.1096	0.1787
Tiglon+Hisat2	14838	34119	0.4349	0.0919	0.1517
Tiglon+Star	15180	33471	0.4535	0.0940	0.1557
StringTie-Merge	16335	39304	0.4156	0.1011	0.1627
StringTie2+MergedAlignmentsnts	16161	42998	0.3759	0.1001	0.1580
StringTie2+Hisat2	14237	33781	0.4214	0.0881	0.1458
StringTie2+Star	14532	34728	0.4185	0.0900	0.1481
Scallop+MergedAlignments	15646	47774	0.3275	0.0969	0.1495
Scallop+Hisat2	14013	38421	0.3647	0.0868	0.1402
Scallop+Star	14869	41511	0.3582	0.0921	0.1465
iPAC+Hisat2	15096	32820	0.4600	0.0935	0.1554
iPAC+Star	15631	33447	0.4673	0.0968	0.1603
TransBorrow+Hisat2	15576	33496	0.4650	0.0964	0.1597
TransBorrow+Star	16244	34511	0.4707	0.1006	0.1657

Table S8. Correct transcripts, assembled transcripts, precision, recall and F-score of the assemblers on the Homo sapiens samples H6 (SRR315323). Related to Figure 3.

Assemblers	Correct transcript	Assembled transcripts	Precision	Recall	F-score
Tiglon+(Hisat2&Star)	13244	39821	0.3326	0.0820	0.1316
Tiglon+Hisat2	10995	40376	0.2723	0.0681	0.1089
Tiglon+Star	11349	41346	0.2745	0.0703	0.1119
StringTie-Merge	12278	39418	0.3115	0.0760	0.1222
StringTie2+MergedAlignmentsnts	12429	46924	0.2649	0.0770	0.1193
StringTie2+Hisat2	10369	35529	0.2918	0.0642	0.1052
StringTie2+Star	11116	36078	0.3081	0.0688	0.1125
Scallop+MergedAlignments	11397	52844	0.2157	0.0706	0.1063
Scallop+Hisat2	9993	43915	0.2276	0.0619	0.0973
Scallop+Star	10653	45408	0.2346	0.0660	0.1030
iPAC+Hisat2	10895	41596	0.2619	0.0675	0.1073
iPAC+Star	11339	44259	0.2562	0.0702	0.1102
TransBorrow+Hisat2	11542	42652	0.2706	0.0715	0.1131
TransBorrow+Star	12130	45772	0.2650	0.0751	0.1170

Table S9. Correct transcripts, assembled transcripts, precision, recall and F-score of the assemblers on the Homo sapiens samples H7 (SRR315334). Related to Figure 3.

Assemblers	Correct transcript	Assembled transcripts	Precision	Recall	F-score
Tiglon+(Hisat2&Star)	16531	39013	0.4237	0.1023	0.1649
Tiglon+Hisat2	14028	36099	0.3886	0.0869	0.1420
Tiglon+Star	14136	33604	0.4207	0.0875	0.1449
StringTie-Merge	15587	42435	0.3673	0.0965	0.1528
StringTie2+MergedAlignmentsnts	15332	43275	0.3543	0.0949	0.1497
StringTie2+Hisat2	13603	35746	0.3805	0.0842	0.1379
StringTie2+Star	14175	36797	0.3852	0.0878	0.1430
Scallop+MergedAlignments	15291	45536	0.3358	0.0947	0.1477
Scallop+Hisat2	13709	37360	0.3669	0.0849	0.1379
Scallop+Star	14527	39837	0.3647	0.0899	0.1443
iPAC+Hisat2	14605	35463	0.4118	0.0904	0.1483
iPAC+Star	14775	33827	0.4368	0.0915	0.1513
TransBorrow+Hisat2	15174	35526	0.4271	0.0939	0.1540
TransBorrow+Star	15698	34958	0.4491	0.0972	0.1598

Table S10. Correct transcripts, assembled transcripts, precision, recall and F-score of the assemblers on the Homo sapiens samples H8 (SRR7536920). Related to Figure 3.

Assemblers	Correct transcript	Assembled transcripts	Precision	Recall	F-score
Tiglon+(Hisat2&Star)	18281	45448	0.4022	0.1132	0.1767
Tiglon+Hisat2	14393	41325	0.3483	0.0891	0.1419
Tiglon+Star	14958	38749	0.3860	0.0926	0.1494
StringTie-Merge	16055	43672	0.3676	0.0994	0.1565
StringTie2+MergedAlignmentsnts	15772	45713	0.3450	0.0976	0.1522
StringTie2+Hisat2	12694	37290	0.3404	0.0786	0.1277
StringTie2+Star	14418	40388	0.3570	0.0893	0.1428
Scallop+MergedAlignments	15752	90791	0.1735	0.0975	0.1249
Scallop+Hisat2	13120	66519	0.1972	0.0812	0.1151
Scallop+Star	14714	75094	0.1959	0.0911	0.1244
iPAC+Hisat2	13715	39768	0.3449	0.0849	0.1363
iPAC+Star	14381	40756	0.3529	0.0890	0.1422
TransBorrow+Hisat2	14037	39490	0.3555	0.0869	0.1397
TransBorrow+Star	15381	40643	0.3784	0.0952	0.1522

Table S11. Correct transcripts, assembled transcripts, precision, recall and F-score of the assemblers on the Mus musculus samples M1 (DRR205674). Related to Figure 4.

Assemblers	Correct transcript	Assembled transcripts	Precision	Recall	F-score
Tiglon+(Hisat2&Star)	14917	28639	0.5209	0.1400	0.2207
Tiglon+Hisat2	13640	28461	0.4793	0.1281	0.2021
Tiglon+Star	13722	28673	0.4786	0.1288	0.2030
StringTie-Merge	14372	31484	0.4565	0.1349	0.2083
StringTie2+MergedAlignmentsnts	14476	35304	0.4100	0.1359	0.2041
StringTie2+Hisat2	13314	28433	0.4683	0.1250	0.1973
StringTie2+Star	13561	29397	0.4613	0.1273	0.1995
Scallop+MergedAlignments	13919	41737	0.3335	0.1307	0.1878
Scallop+Hisat2	13023	32912	0.3957	0.1223	0.1868
Scallop+Star	13204	34923	0.3781	0.1240	0.1867
iPAC+Hisat2	13453	25563	0.5263	0.1263	0.2037
iPAC+Star	13480	26358	0.5114	0.1265	0.2029
TransBorrow+Hisat2	13852	26757	0.5177	0.1300	0.2079
TransBorrow+Star	14055	27955	0.5028	0.1319	0.2090

Table S12. Correct transcripts, assembled transcripts, precision, recall and F-score of the assemblers on Mus musculus samples M2 (DRR205677). Related to Figure 4.

Assemblers	Correct transcript	Assembled transcripts	Precision	Recall	F-score
Tiglon+(Hisat2&Star)	14450	28014	0.5158	0.1357	0.2148
Tiglon+Hisat2	13199	27749	0.4757	0.1239	0.1966
Tiglon+Star	13285	27948	0.4753	0.1247	0.1976
StringTie-Merge	13985	30655	0.4562	0.1313	0.2039
StringTie2+MergedAlignmentsnts	14206	34737	0.4090	0.1334	0.2011
StringTie2+Hisat2	12993	27827	0.4669	0.1220	0.1934
StringTie2+Star	13226	28714	0.4606	0.1242	0.1956
Scallop+MergedAlignments	13577	40626	0.3342	0.1275	0.1845
Scallop+Hisat2	12669	31830	0.3980	0.1189	0.1831
Scallop+Star	12853	33470	0.3840	0.1207	0.1836
iPAC+Hisat2	12926	24877	0.5196	0.1213	0.1967
iPAC+Star	12968	25438	0.5098	0.1217	0.1965
TransBorrow+Hisat2	13392	26083	0.5134	0.1257	0.2020
TransBorrow+Star	13607	27117	0.5018	0.1277	0.2036

Table S13. Correct transcripts, assembled transcripts, precision, recall and F-score of the assemblers on the Mus musculus samples M3 (ERR3320855). Related to Figure 4.

Assemblers	Correct transcript	Assembled transcripts	Precision	Recall	F-score
Tiglon+(Hisat2&Star)	17683	33534	0.5273	0.1660	0.2525
Tiglon+Hisat2	15418	32140	0.4797	0.1447	0.2224
Tiglon+Star	15874	31469	0.5044	0.1490	0.2301
StringTie-Merge	16879	38046	0.4436	0.1585	0.2335
StringTie2+MergedAlignmentsnts	16844	44361	0.3797	0.1581	0.2233
StringTie2+Hisat2	15066	34519	0.4365	0.1414	0.2136
StringTie2+Star	15457	35963	0.4298	0.1451	0.2170
Scallop+MergedAlignments	16377	52867	0.3098	0.1537	0.2055
Scallop+Hisat2	14824	44848	0.3305	0.1392	0.1959
Scallop+Star	15311	47167	0.3246	0.1437	0.1992
iPAC+Hisat2	15332	29461	0.5204	0.1439	0.2255
iPAC+Star	15860	29737	0.5333	0.1489	0.2328
TransBorrow+Hisat2	15832	31491	0.5027	0.1486	0.2294
TransBorrow+Star	16519	32057	0.5153	0.1551	0.2384

Table S14. Correct transcripts, assembled transcripts, precision, recall and F-score of the assemblers on the Mus musculus samples M4 (ERR3320871). Related to Figure 4.

Assemblers	Correct transcript	Assembled transcripts	Precision	Recall	F-score
Tiglon+(Hisat2&Star)	17560	34815	0.5044	0.1649	0.2485
Tiglon+Hisat2	15343	33378	0.4597	0.1440	0.2193
Tiglon+Star	15827	33672	0.4700	0.1486	0.2258
StringTie-Merge	16450	39114	0.4206	0.1544	0.2259
StringTie2+MergedAlignmentsnts	16431	52801	0.3112	0.1543	0.2063
StringTie2+Hisat2	14650	36566	0.4006	0.1375	0.2048
StringTie2+Star	15073	38281	0.3937	0.1415	0.2082
Scallop+MergedAlignments	15808	59178	0.2671	0.1484	0.1908
Scallop+Hisat2	14466	50473	0.2866	0.1358	0.1843
Scallop+Star	14777	52808	0.2798	0.1387	0.1855
iPAC+Hisat2	15266	31162	0.4899	0.1433	0.2218
iPAC+Star	15799	32240	0.4900	0.1483	0.2277
TransBorrow+Hisat2	15684	33558	0.4674	0.1472	0.2239
TransBorrow+Star	16331	34897	0.4680	0.1533	0.2310

Table S15 Correct transcripts on simulated dataset with low, middle or high expression levels. Related to Figure 2.

Assemblers	Low expressed	Middle expressed	High expressed
Tiglon+(Hisat2&Star)	1236	6279	15413
Tiglon+Hisat2	903	4933	14998
Tiglon+Star	1002	5164	14723
StringTie-Merge	950	5133	14547
StringTie2+MergedAlignments	1038	5008	14608
StringTie2+Hisat2	669	3894	14237
StringTie2+Star	730	4345	14192
Scallop+MergedAlignments	782	4747	14748
Scallop+Hisat2	490	3891	14405
Scallop+Star	595	4399	14469

Table S16 Correct transcripts on the Homo sapiens sample H1 (SRR307911) with low, middle or high expression levels. Related to Figure 3.

Assemblers	Low expressed	Middle expressed	High expressed
Tiglon+(Hisat2&Star)	1008	3606	11343
Tiglon+Hisat2	729	2681	10027
Tiglon+Star	750	2824	10336
StringTie-Merge	794	3243	11159
StringTie2+MergedAlignments	777	3130	11209
StringTie2+Hisat2	570	2408	10214
StringTie2+Star	616	2708	10601
Scallop+MergedAlignments	684	2807	11104
Scallop+Hisat2	528	2314	10260
Scallop+Star	550	2608	10614

Table S17 Correct transcripts on the Homo sapiens sample H2 (SRR387662) with low, middle or high expression levels. Related to Figure 3.

Assemblers	Low expressed	Middle expressed	High expressed
Tiglon+(Hisat2&Star)	1021	3735	10234
Tiglon+Hisat2	736	2956	9198
Tiglon+Star	722	2796	9114
StringTie-Merge	833	3281	9857
StringTie2+MergedAlignments	781	3021	9717
StringTie2+Hisat2	609	2621	9226
StringTie2+Star	672	2762	9265
Scallop+MergedAlignments	725	2884	10032
Scallop+Hisat2	607	2618	9427
Scallop+Star	641	2655	9560

Table S18 Correct transcripts on the Homo sapiens sample H3 (SRR10517380) with low, middle or high expression levels. Related to Figure 3.

Assemblers	Low expressed	Middle expressed	High expressed
Tiglon+(Hisat2&Star)	1336	4685	13346
Tiglon+Hisat2	1065	3848	12640
Tiglon+Star	1122	3950	12441
StringTie-Merge	903	3815	12573
StringTie2+MergedAlignments	893	3639	12608
StringTie2+Hisat2	707	3080	12237
StringTie2+Star	714	3153	11997
Scallop+MergedAlignments	722	3254	12732
Scallop+Hisat2	603	2976	12326
Scallop+Star	630	3091	12411

Table S19 Correct transcripts on the Homo sapiens sample H4 (ERR2403203) with low, middle or high expression levels. Related to Figure 3.

Assemblers	Low expressed	Middle expressed	High expressed
Tiglon+(Hisat2&Star)	1257	4933	12737
Tiglon+Hisat2	949	3901	12096
Tiglon+Star	1017	4184	12045
StringTie-Merge	909	4087	12064
StringTie2+MergedAlignments	859	3808	11983
StringTie2+Hisat2	701	3341	11833
StringTie2+Star	674	3353	11522
Scallop+MergedAlignments	737	3768	12410
Scallop+Hisat2	611	3373	12125
Scallop+Star	633	3594	12094

Table S20 Correct transcripts on the Homo sapiens sample H5 (SRR307903) with low, middle or high expression levels. Related to Figure 3.

Assemblers	Low expressed	Middle expressed	High expressed
Tiglon+(Hisat2&Star)	1081	3952	11888
Tiglon+Hisat2	772	2894	10555
Tiglon+Star	805	3073	10698
StringTie-Merge	827	3334	11529
StringTie2+MergedAlignments	828	3267	11492
StringTie2+Hisat2	591	2570	10599
StringTie2+Star	624	2683	10773
Scallop+MergedAlignments	638	3014	11589
Scallop+Hisat2	480	2449	10726
Scallop+Star	515	2809	11165

Table S21 Correct transcripts on the Homo sapiens sample H6 (SRR315323) with low, middle or high expression levels. Related to Figure 3.

Assemblers	Low expressed	Middle expressed	High expressed
Tiglon+(Hisat2&Star)	874	2569	9123
Tiglon+Hisat2	600	1826	8090
Tiglon+Star	690	2092	8039
StringTie-Merge	619	2165	8974
StringTie2+MergedAlignments	626	2229	9094
StringTie2+Hisat2	429	1536	8015
StringTie2+Star	498	1830	8411
Scallop+MergedAlignments	532	1875	8628
Scallop+Hisat2	393	1483	7815
Scallop+Star	432	1731	8159

Table S22 Correct transcripts on the Homo sapiens sample H7 (SRR315334) with low, middle or high expression levels. Related to Figure 3.

Assemblers	Low expressed	Middle expressed	High expressed
Tiglon+(Hisat2&Star)	909	3790	11072
Tiglon+Hisat2	653	2864	9919
Tiglon+Star	668	2905	9980
StringTie-Merge	714	3381	10869
StringTie2+MergedAlignments	678	3202	10892
StringTie2+Hisat2	527	2537	10067
StringTie2+Star	560	2788	10367
Scallop+MergedAlignments	646	3085	11070
Scallop+Hisat2	457	2577	10250
Scallop+Star	543	2882	10646

Table S23 Correct transcripts on the Homo sapiens sample H8 (SRR7536920) with low, middle or high expression levels. Related to Figure 3.

Assemblers	Low expressed	Middle expressed	High expressed
Tiglon+(Hisat2&Star)	1191	4000	12268
Tiglon+Hisat2	797	2628	10423
Tiglon+Star	781	2936	10658
StringTie-Merge	776	3176	11488
StringTie2+MergedAlignments	733	2992	11502
StringTie2+Hisat2	506	2026	9764
StringTie2+Star	643	2608	10696
Scallop+MergedAlignments	630	2951	11686
Scallop+Hisat2	456	2176	10128
Scallop+Star	550	2728	10979

Table S24 Correct transcripts on the Mus musculus sample M1 (DRR205674) with low, middle or high expression levels. Related to Figure 4.

Assemblers	Low expressed	Middle expressed	High expressed
Tiglon+(Hisat2&Star)	740	3800	9998
Tiglon+Hisat2	589	3116	9601
Tiglon+Star	655	3375	9374
StringTie-Merge	565	3527	9921
StringTie2+MergedAlignments	602	3531	9991
StringTie2+Hisat2	421	2899	9715
StringTie2+Star	474	3134	9657
Scallop+MergedAlignments	444	3192	10017
Scallop+Hisat2	325	2745	9736
Scallop+Star	351	2957	9669

Table S25 Correct transcripts on the Mus musculus sample M2 (DRR205677) with low, middle or high expression levels. Related to Figure 4.

Assemblers	Low expressed	Middle expressed	High expressed
Tiglon+(Hisat2&Star)	676	3646	9717
Tiglon+Hisat2	518	3008	9323
Tiglon+Star	572	3212	9170
StringTie-Merge	526	3371	9684
StringTie2+MergedAlignments	565	3435	9825
StringTie2+Hisat2	403	2770	9501
StringTie2+Star	431	3005	9469
Scallop+MergedAlignments	435	3038	9826
Scallop+Hisat2	298	2582	9539
Scallop+Star	324	2788	9494

Table S26 Correct transcripts on the Mus musculus sample M3 (ERR3320855) with low, middle or high expression levels. Related to Figure 4.

Assemblers	Low expressed	Middle expressed	High expressed
Tiglon+(Hisat2&Star)	781	4513	12020
Tiglon+Hisat2	556	3484	11080
Tiglon+Star	630	3824	11127
StringTie-Merge	623	4045	11889
StringTie2+MergedAlignments	591	3952	12018
StringTie2+Hisat2	451	3151	11224
StringTie2+Star	470	3373	11370
Scallop+MergedAlignments	476	3585	12102
Scallop+Hisat2	339	3011	11283
Scallop+Star	366	3280	11464

Table S27 Correct transcripts on the Mus musculus sample M4 (ERR3320871) with low, middle or high expression levels. Related to Figure 4.

Assemblers	Low expressed	Middle expressed	High expressed
Tiglon+(Hisat2&Star)	908	4157	12097
Tiglon+Hisat2	645	3216	11173
Tiglon+Star	754	3546	11220
StringTie-Merge	649	3605	11861
StringTie2+MergedAlignments	626	3526	11959
StringTie2+Hisat2	457	2798	11134
StringTie2+Star	507	3030	11289
Scallop+MergedAlignments	463	3088	12032
Scallop+Hisat2	357	2699	11218
Scallop+Star	379	2831	11376
